# Metabolomic disorders: confirmed presence of potentially treatable abnormalities in patients with treatment refractory depression and suicidal behavior

**DOI:** 10.1017/S0033291722003233

**Published:** 2023-10

**Authors:** Lisa A. Pan, Anna Maria Segreti, Joseph Wrobleski, Annie Shaw, Keith Hyland, Marion Hughes, David N. Finegold, Robert K. Naviaux, David A. Brent, Jerry Vockley, David G. Peters

**Affiliations:** 1 University of Pittsburgh, School of Medicine, Pittsburgh, PA 15213, USA; 2 New Hope Molecular, Pittsburgh, PA 15228, USA; 3 University of Pittsburgh, Graduate School of Public Health, Pittsburgh, PA 15261, USA; 4 Panomics Mental Health Initiative, Pittsburgh, PA 15228, USA; 5 Medical Neurogenetics Laboratory, Atlanta, Georgia 30342, USA; 6 University of California at San Diego, School of Medicine, San Diego, California 92103, USA; 7 Magee-Womens Research Institute, Pittsburgh, PA 15213, USA

**Keywords:** Cerebral folate deficiency, metabolomics, treatment refractory depression, tetrahydrobiopterin, suicide

## Abstract

**Background:**

Refractory depression is a devastating condition with significant morbidity, mortality, and societal cost. Approximately 15% of patients with major depressive disorder are refractory to currently available treatments. We hypothesized metabolic abnormalities contributing to treatment refractory depression are associated with distinct findings identifiable in the cerebrospinal fluid (CSF). Our hypothesis was confirmed by a previous small case-controlled study. Here we present a second, larger replication study.

**Methods:**

We conducted a case-controlled, targeted, metabolomic evaluation of 141 adolescent and adult patients with well-characterized history of depression refractory to three maximum-dose, adequate-duration medication treatments, and 36 healthy controls. Plasma, urine, and CSF metabolic profiling were performed by coupled gas chromatography/mass spectrometry, and high-performance liquid chromatography, electrospray ionization, tandem mass spectrometry.

**Results:**

Abnormalities were identified in 67 of 141 treatment refractory depression participants. The CSF abnormalities included: low cerebral folate (*n* = 20), low tetrahydrobiopterin intermediates (*n* = 11), and borderline low-tetrahydrobiopterin intermediates (*n* = 20). Serum abnormalities included abnormal acylcarnitine profile (*n* = 12) and abnormal serum amino acids (*n* = 20). Eighteen patients presented with two or more abnormal metabolic findings. Sixteen patients with cerebral folate deficiency and seven with low tetrahydrobiopterin intermediates in CSF showed improvement in depression symptom inventories after treatment with folinic acid and sapropterin, respectively. No healthy controls had a metabolite abnormality.

**Conclusions:**

Examination of metabolic disorders in treatment refractory depression identified an unexpectedly large proportion of patients with potentially treatable abnormalities. The etiology of these abnormalities and their potential roles in pathogenesis remain to be determined.

## Background

Major depressive disorder is the second-leading cause of disability world-wide and affects an estimated 350 million people (Souery, Papkostas, & Trivedi, 2006). Although the treatment of depression has advanced, an estimated 15% of depressed individuals do not respond despite adequate pharmacotherapy, psychotherapy, and even electroconvulsive therapy (Anonymous, 2014). Current predictors of treatment response for depression, such as severity, chronicity, or exposure to adverse life events, are non-specific and without clear therapeutic implications in treatment refractory depression. Despite new medications, the clinical management of treatment refractory depression has not advanced significantly in the past two decades and, unfortunately, the suicide rate in the United States has been increasing (Centers for Disease Control and Prevention, 2022). Recent emphasis on treatment refractory depression has focused on ketamine and neuromodulatory treatments, such as deep brain stimulation, which, while promising, require neurosurgical intervention. We propose a novel diagnostic and therapeutic approach to treatment refractory depression based on a targeted analysis of metabolites in blood, urine, and cerebral spinal fluid (CSF).

Metabolomics is the study of patterns of metabolic intermediates to characterize dysfunctional metabolic pathways potentially related to clinical symptoms. This approach has previously contributed to our understanding of the pathophysiology of depression. For example, over 3 decades ago, decreased 5-hydroxyindoleacetic acid (5-HIAA) (the main metabolite of serotonin) in CSF of depressed patients was shown to convey a markedly increased risk for suicide (Asberg, Traskman, & Thoren, 1976). This work helped to spur the development of selective serotonin reuptake inhibitors (SSRIs), which are now widely used for the treatment of depression (Asberg & Forslund, 2000; Axelrod, 1972). In addition, neuropsychiatric symptoms are common manifestations of many inborn errors of metabolism. Adolescent and adult neuropsychiatric symptoms, possibly caused by metabolic abnormalities, are also a common comorbidity in both primary (Anglin, Garside, Tarnopolsky, Mazurek, & Rosebush, 2012) and secondary forms of mitochondrial dysfunction (Morris & Berk, 2015).

We previously reported a 19-year-old male patient, with unremitting treatment refractory depression and repeated suicide attempts who exhibited deficient tetrahydrobiopterin intermediates in CSF and was responsive to replacement with the tetrahydrobiopterin analog sapropterin (Pan et al., 2011). CSF testing of five additional adolescent patients with treatment refractory depression revealed that three had low CSF 5-methyltetrahydrofolate (5-MTHF) and normal blood folate levels, indicating cerebral folate deficiency (Hyland, Shoffner, & Heales, 2010). A follow up case-control study revealed potentially treatable metabolite abnormalities in 21 of 33 participants with treatment refractory depression and in none of the healthy controls (Pan et al., 2016). None of the conventional clinical or research diagnostic or therapeutic approaches would have identified the source of these patients' difficulties, nor would they have suggested the subsequent successful treatment strategies.

In this report, we describe the systematic evaluation of 141 patients with treatment-refractory depression for primary and secondary disorders of central nervous system (CNS) metabolism. Targeted studies of CSF and peripheral samples (blood and urine) identified patients with abnormalities in the monoamine neurotransmitter synthesis pathway, as well other metabolomic abnormalities leading to apparent isolated CNS dysfunction with depression. We hypothesized that we would replicate our earlier findings, indicating that the incidence of metabolic abnormalities contributing to treatment refractory depression would be significantly greater in affected patients than in healthy controls. Broader identification of such metabolic disorders could transform psychiatric practice, particularly in severe depression, when standard treatments are ineffective.

## Methods

### Sample characteristics

Participants aged 14 to 70 years with depression unresponsive to known treatments (at least three maximum dose medication trials, each for at least 6 weeks) were recruited by advertisement through the Clinical and Translational Science Institute Registry at University of Pittsburgh or by clinical referral. All adult participants provided informed consent; adolescent participants provided informed assent for involvement in the study, with parents providing informed consent. Participants were compared with young adult healthy controls with no personal or first-degree relative history of psychiatric disorder or suicidal behavior. Adolescent healthy controls were excluded from participation because a lumbar puncture conferred greater than minimal risk. This study was approved by the Institutional Review Board of the University of Pittsburgh.

Healthy controls (*n* = 36) and treatment refractory depression participants (*n* = 141) were enrolled. Two healthy controls and one affected participant were excluded from the CSF analysis due to oxidation of their CSF sample.

Participants were assessed with a structured psychiatric interview, including the Family Interview for Genetics Studies (NIMH, 1992), at the time of referral to characterize depression course, comorbidity, family history, and history of trauma, psychosis, and anxiety. Treatment refractory depression status was confirmed with the Antidepressant Treatment History Questionnaire (Fava, 2003). The Beck Depression Inventory (BDI) (Beck, Ward, Mendelson, Mock, & Erbaugh, 1961) and Suicide Ideation Questionnaire (Reynolds, 1987) were collected for all participants and the Child Depression Rating Scale-Revised (Poznanski & Mokros, 1996) was collected from clinical assessment in adolescents. Patients remained on current medications and other current treatments during the study.

### Study procedures

For each participant, the research staff and principal investigator (a) completed a clinical and structured psychiatric interview, (b) obtained self-report symptom questionnaires, and (c) arranged for study laboratory testing. Assessment consisted of a psychiatric interview, review of records, and administered self-reports at intake [characterized depression course (Beck Depression Inventory) {BDI}], suicidal ideation and behavior [Suicidal Ideation Questionnaire, Columbia Suicide History Form (Posner, Oquendo, Gould, Stanley, & Davies, 2007), and Beck Suicide Ideation Scale (Beck, Schuyler, & Herman, 1974), comorbidity (anxiety, psychosis, substance use, attention disorders) (DSM 5 Cross Cutting Symptom Inventory) (Clarke & Kuhl, 2014), family history (Family Interview for Genetic Studies and 3 generation pedigree) (NIMH, 1992)]. A neurologic examination was completed by the principal investigator (L.P.). Analysis of blood and CSF began promptly after collection.

Plasma and urine testing were performed by the Clinical Biochemical Genetics and Clinical Chemistry Laboratories of University of Pittsburgh Medical Center (UPMC) per their standard protocols including gas chromatography-mass spectrometry (GC-MS) of urine, and liquid chromatography-tandem mass spectrometry (LC-MS/MS) and high-pressure liquid chromatography-MS/MS (HPLC-MS/MS) profiling of blood to examine known groups of metabolites contributing to depression. A lumbar puncture for CSF collection was performed under fluoroscopic guidance by Interventional Radiology at UPMC, and samples were analyzed as for blood and urine by the Clinical Biochemical Genetics and Clinical Chemistry Laboratories of UPMC and Medical Neurogenetics, Inc. (Atlanta, GA). If a specific abnormality was suspected based on the initial testing, the patient was referred to a biochemical geneticist for additional confirmatory testing. Upon receipt of results of testing, a follow-up appointment was scheduled for every affected participant to review results and provide additional referrals if needed. Participants with treatment refractory depression returned for a second appointment to review results. At this appointment, BDI, Suicidal Ideation Questionnaire, and DSM 5 Symptom Inventory were repeated. Participants for whom a novel treatment was available were re-contacted at least 6 weeks after the start of treatment to determine the outcome with repeated BDI, Suicidal Ideation Questionnaire, and DSM 5 Symptom Inventory.

Statistical analyses were completed in SPSS v.21. We employed independent *t* tests to examine the differences between treatment refractory depression participants and healthy controls in depression severity (BDI), suicidal ideation (Suicidal Ideation Questionnaire), and age. A χ^2^ goodness of fit test was calculated to compare the distribution of male to female participants in each group. *t* tests were performed between pre- and post-treatment BDI and Suicidal Ideation Questionnaire scores among the cerebral folate deficient participants.

## Results

### Demographics and clinical assessment

Table 1. The treatment refractory depression and healthy control groups were similar with respect to age and sex, and ethnicity.

**Table 1.** Demographics

	Healthy controls (36 total)	Depressed (141 total)
Average age (median, range)	24.64 (±5.65)	28.20(±11.89)
Sex		
Number of males (%)	15 (42%)	54 (38%)
Number of females (%)	21 (58%)	87 (62%)
Race		
African American	4	1
Asian	7	6
Caucasian	22	130
Multiple races	3	4
Ethnicity		
Hispanic	1	5
Not Hispanic	35	136

### Characterization of depression

Table 2 and online Supplementary Table S3. Treatment refractory depression participants reported longstanding, unremitting, or recurrent depression with an average of 2.45 episodes (s.d. = 7.75), with average longest duration of episode without symptom remission having a mean of 640.12 weeks (s.d. 578.65 weeks). Mean age of onset of depressive symptoms in treatment refractory depression participants was 12.35 years old (s.d. 5.21). The BDI was much higher in the treatment refractory depression participants than in the healthy controls [40.11 (s.d. = 20.64) *v.* 1.42 (s.d. = 2.29], [(*t*(175) = 11.18), *p* < 0.001]. The treatment refractory depression group also showed much higher Suicidal Ideation Questionnaire scores than did the healthy controls [30.91 (s.d. = 9.92) *v.* 1.81 (s.d. = 2.16], [(*t*(175) = 17.39), *p* < 0.001]. Sixty-eight treatment refractory depression (48%) participants reported a history of at least one suicide attempt.

**Table 2.** Summary of characterization of depression and treatment results

	Healthy controls (*N* = 36)	Low cerebral folate (*N* = 20)	Low tetrahydrobiopterin intermediates (*n* = 11)	Borderline-low tetrahydrobiopterin intermediates (*N* = 20)	Abnormal acyl-carnitine profile (*N* = 12)	Abnormal serum amino acids (*N* = 20)
Age (Mean ± s.d.)	24.64 (±5.65)	27.95 (±10.23)	29.18 (±10.69)	33.8 (±14.07)	29.25 (±10.15)	33.55 (±10.75)
Number of males (%)	15 (42%)	7 (35%)	3 (27%)	6 (30%)	7 (58%)	10 (50%)
Number of females (%)	21 (58%)	13 (65%)	8 (73%)	14 (70%)	5 (42%)	10 (50%)
Number of medication trials (Mean ± s.d.)	0	7.35 (±3.01)	8.55 (±2.58)	8.7 (±2.03)	7.42 (±2.71)	8.5 (±2.19)
Number of suicide attempts (Mean ± s.d.)	0	1.6 (±2.6)	1.55 (±2.95)	1.3 (±2.34)	1.33 (±2.87)	0.8 (±1.47)
Number of depressed episodes (Mean ± s.d.)	0	10.95 (±28.81)	1.36 (±.92)	1.7 (±1.49)	8.67 (±25.62)	6.75 (±22.01)
Number treated with ECT (%)	0	8 (40%)	6 (54%)	5 (25%)	2 (17%)	5 (25%)
Number with bipolar disorder (%)	0	2 (10%)	2 (18%)	8 (40%)	3 (25%)	6 (30%)
Number with family history of MDD (%)	0	16 (80%)	8 (73%)	15 (75%)	8 (67%)	17 (85%)
Number with family history of bipolar disorder (%)	0	7 (35%)	2 (18%)	3 (15%)	3 (25%)	4 (20%)
Initial SIQ (Mean ± s.d.)	1.81 (±2.16)	Out of 16 with follow-up scores: 40.31 (±20.15)	Out of 7 with follow-up scores: 60.14 (+14.17)	Out of 5 with follow-up scores: 40.6 (±24.94)	44.67 (±22.21)	36 (±17.35)
Initial BDI (Mean ± s.d.)	1.42 (±2.29)	Out of 16 with follow-up scores: 30.94 (±9.82)	Out of 7 with follow-up scores: 42.86 (±11.08)	Out of 5 with follow-up scores: 30 (±13.45)	31.25 (±8.51)	30.9 (±9.37)
Post-treatment SIQ (Mean ± s.d.)	N/A	Out of 16 with follow-up scores: 22.88 (±16.94)	Out of 7 with follow-up scores: 38.29 (±21.72)	Out of 5 with follow-up scores: 20 (±19.24)	N/A	N/A
Post-treatment BDI (Mean ± s.d.)	N/A	Out of 16 with follow-up scores: 18.5 (±12.09)	Out of 7 with follow-up scores: 27.71 (±19.05)	Out of 5 with follow-up scores: 15.6 (±13.97)	N/A	N/A
Time between initial and post-treatment reports (Mean ± s.d., weeks)	N/A	Out of 16 with follow-up scores: 20.31(±20.84)	Out of 7 with follow-up scores: 39.71 (±19.47)	Out of 5 with follow-up scores: 22.4 (±18.04)	N/A	N/A

MDD, major depressive disorder; ECT, electroconvulsive therapy; SIQ, Suicide Ideation Questionnaire; BDI, Beck Depression Inventory.

Of the 141 participants with treatment refractory depression, there was a positive family history for at least one first degree relative of any disorder (83%), depression (72%), and suicide attempts (17%) on the Family Interview for Genetic Studies. Healthy controls had no personal or first-degree relative history of psychiatric disorder or suicidal behavior (by definition). On neurologic exam, neither treatment refractory depression participants nor healthy controls had abnormalities, except for one treatment refractory depression patient with benign essential tremor.

### Metabolic targeted clinical studies

Table 2 and online Supplementary Table S4. We identified evidence of metabolomic abnormalities in 67 of 141 participants (48%) with treatment refractory depression (Table 2 and online Supplementary Table S4). CFD [CSF 5-methyltetrahydrofolate (5-MTHF) deficiency], a metabolic abnormality in which serum folate is normal, but CSF 5-MTHF is low (*<40 nmol/L*), was present in 20 participants (14%) (online Supplementary Fig. S3). Low tetrahydrobiopterin intermediates in CSF (*<10 nmol/l*) were present in 11 participants (7%), and borderline low tetrahydrobiopterin intermediates in CSF (*<12 nmol/l*) were present in 20 participants (14%) (online Supplementary Fig.va S4). Three participants with CFD were also found to have low tetrahydrobiopterin metabolites in CSF, as seen in our previously published case who responded to treatment with sapropterin (Pan et al., 2011). Abnormal serum acylcarnitine profiles were identified in 12 participants (8%). Abnormal serum amino acids were found in 20 participants (14%). Eighteen patients (13%) presented with two or more abnormal metabolic findings. Interleukin-6 was measured in 136 patients with MDD, (five patients did not have IL-6 testing due to sample availability) with mean IL-6 = 3.15 (±9.589) compared to 25 HC (11 controls did not have IL-6 testing) with mean IL-6 = 3.15 (±0.537).

### Treatment

**Fig. 1.**
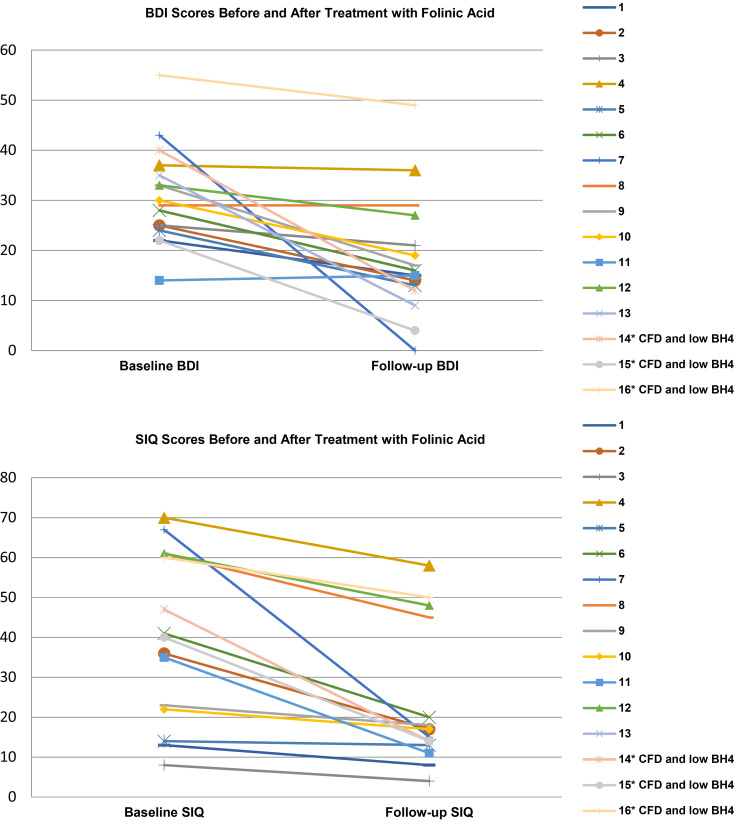
Symptom inventory outcomes in patients with cerebral folate deficiency before and after treatment with folinic acid. Sixteen participants with CFD, and treated with folinic acid, showed reductions in SIQ scores, and 7 out of 8 participants showed reductions in BDI scores (1 participant showed an increased BDI score by 1 point following treatment). In patients with CFD, average BDI score decreased from 30.94 (s.d. 9.82) to 18.5 (s.d. 12.09), *t* tests revealed that the change was statistically significant, *t* = 3.83, *p* < 0.003. Average Suicidal Ideation Questionnaire score decreased from 40.31 (s.d. 20.15) to 22.88 (s.d. 16.94), *t* tests revealed that the change was statistically significant, *t* = 3.03, *p* < 0.01.

20 patients with low CSF 5MTHF were offered treatment with folinic acid, three were non-compliant with the folinic acid treatment, and one was lost to follow-up. Sixteen were provided treatment with folinic acid 1–2 mg/kg for at least six weeks (range 6–28 weeks), and all showed reduction in symptom inventories at follow-up along with taking their pre-evaluation treatment regimen. Out of the 16 patients with CFD who were treated with folinic acid, three had a co-morbid biochemical finding of low CSF tetrahydrobiopterin metabolites in their CSF and were also treated with sapropterin 20 mg/kg, three had an abnormal serum amino acid finding, one patient had an abnormal acylcarnitine profile, and nine did not have a co-morbid biochemical finding. All 16 participants with CFD, and treated with folinic acid, showed reductions in SIQ scores, and 15 out of 16 participants showed reductions in BDI scores (1 participant showed an increased BDI score by 1 point following treatment). In patients with CFD, average BDI score decreased from 30.94 (s.d. 9.82) to 18.5 (s.d. 12.09), *t* tests revealed that the change was statistically significant, *t* = 3.83,df = *p* < 0.003. Average Suicidal Ideation Questionnaire score decreased from 40.31 (s.d. 20.15) to 22.88 (s.d. 16.94), *t* tests revealed that the change was statistically significant, *t* = 3.03, df *p* < 0.01. (Table 2, Fig. 1, and online Supplementary Table S4).

**Fig. 2.**
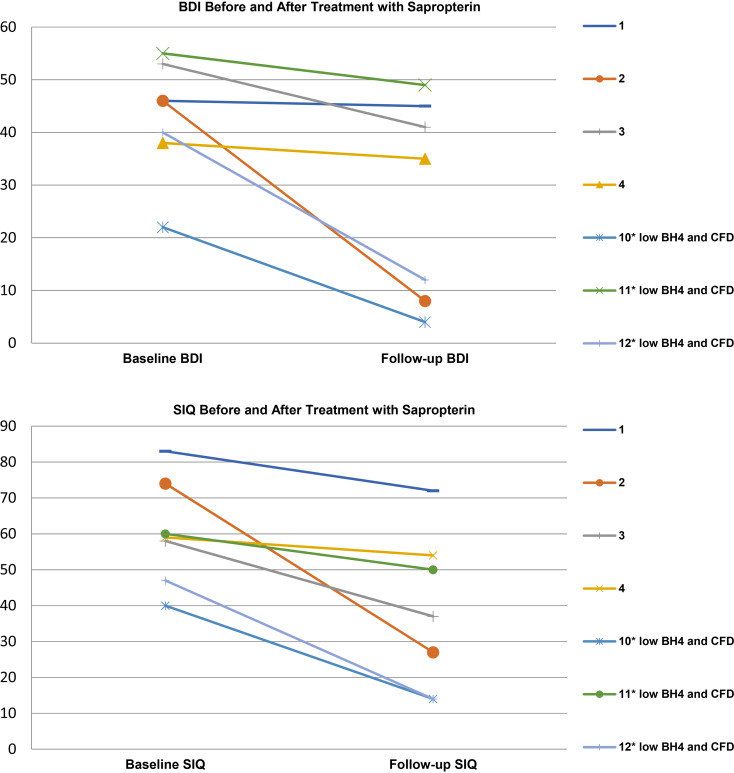
Symptom inventory outcomes in patients with tetrahydrobiopterin deficiency before and after treatment with sapropterin. Seven patients with low tetrahydrobiopterin levels in their CSF who were treated with sapropterin 20 mg/kg showed significant reductions in their follow-up SIQ and BDI scores. Average BDI scores decreased from 42.86 (s.d. 11.08) to 27.71 (s.d. 19.05), although, *t* tests revealed that the change was not statistically significant, *t* = 1.85, *p* < 0.09. Average Suicidal Ideation Questionnaire score decreased from 60.14 (s.d. 14.71) to 38.29 (s.d. 21.72), *t* tests revealed that the change was statistically significant, *t* = 2.25, *p* < 0.04.

Of the 11 patients with low tetrahydrobiopterin intermediates in CSF (<10 nmol/l), two were non-compliant with sapropterin treatment and two were lost to follow-up. Seven were provided treatment with sapropterin 20 mg/kg for at least six weeks (range 6–60 weeks), and all showed reductions in symptom inventories at follow-up along with taking their pre-evaluation treatment regimen. Out of the seven patients with low tetrahydrobiopterin who were treated with sapropterin, three were also treated for CFD with folinic acid 1–2 mg/kg, one patient had abnormal serum amino acids, and three did not have a co-morbid biochemical finding. All seven participants with low tetrahydrobiopterin, who were treated with sapropterin and completed a follow-up appointment, showed reductions in SIQ and BDI scores. Average BDI scores decreased from 42.86 (s.d. 11.08) to 27.71 (s.d. 19.05), although, *t* tests revealed that the change was not statistically significant, *t* = 1.85, *p* < 0.09. Average Suicidal Ideation Questionnaire score decreased from 60.14 (s.d. 14.71) to 38.29 (s.d. 21.72), *t* tests revealed that the change was statistically significant, *t* = 2.25, *p* < 0.04. (Table 2, Fig. 2, and online Supplementary Table S4).

Of the 20 patients with borderline-low tetrahydrobiopterin intermediates in CSF (<12 nmol/l), fifteen were non-compliant with the sapropterin treatment. Five were treated with sapropterin 20 mg/kg for at least six weeks (range 6–60 weeks), and all showed reduction in symptom inventories at follow-up along with taking their pre-evaluation treatment regimen. Out of the 5 patients with borderline-low tetrahydrobiopterin who were treated with sapropterin, two patients had an abnormal serum amino acid finding, and three did not have a co-morbid biochemical finding. All five participants with borderline-low tetrahydrobiopterin, who were treated with sapropterin and completed a follow-up appointment, showed reductions in SIQ and BDI scores. Average BDI score decreased from 30 (s.d. 13.45) to 15.6 (s.d. 13.97), although, *t* tests revealed that the change was not statistically significant, *t* = 1.62, *p* < 0.13. Average Suicidal Ideation Questionnaire score decreased from 40.6 (s.d. 24.93) to 20 (s.d. 19.24), although, *t* tests revealed that the change was not statistically significant, *t* = 1.44, *p* < 0.18 (Table 2 and online Supplementary Table S4).

## Conclusions

Our initial case control study (Pan et al., 2016) and this replication CNS-specific metabolomic survey are the first to systematically evaluate abnormalities of neurotransmitter, vitamin, pterin, and energy metabolism in peripheral and CSF samples from patients with isolated psychiatric symptoms in the absence of primary neurologic symptoms. The studies were spurred by our initial identification of a young man with deficient tetrahydrobiopterin and response to treatment with sapropterin, followed by discovery of CFD in 3 of 5 additional patients (Pan et al., 2011). In this case-control study of individuals with treatment refractory depression, 48% of patients (67 of 141) had metabolomic abnormalities. Results were determined based on established normal clinical ranges for all tests reported.

Twenty patients had cerebral folate deficiency, including sixteen with reductions in BDI and Suicidal Ideation Questionnaire scores following treatment with folinic acid. In all cases patients had normal serum metabolites associated with folate pathways, and therefore would have eluded conventional diagnostic approaches without a more comprehensive evaluation.

We were unable to determine whether the abnormalities identified were due to a primary genetic disorder in a metabolic pathway or a secondary abnormality of indirect etiology (e.g. acquired injuries to the CNS from infections, inflammation of unknown cause, autoimmune disease, asphyxia, or epilepsy) typically seen with other obvious systemic manifestations (Serrano, Perez-Duenas, Montoya, Ormazabal, & Artuch, 2012). The abnormalities identified might also be sequelae of unremitting depression. CFD is a CNS specific syndrome characterized by low 5-MTHF in CSF and normal folate in plasma (Pan & Vockley, 2013; Sedel, 2013). It may be caused primarily by gene abnormalities, may be secondarily reduced in any metabolic defect that increases use of methyl groups, or caused by changes in transport across the blood brain barrier. Secondary CFD is also seen in disorders of the mitochondrial respiratory chain such as Alpers syndrome (Cohen, Chinnery, & Copeland, 2010), Kearns-Sayre syndrome (Quijada-Fraile et al., 2014), and others (Garcia-Cazorla et al., 2008). Folate is involved in nearly 100 metabolic reactions (Bottiglieri et al., 1992), including the purine synthetic pathway, contributing to impaired tetrahydrobiopterin, serotonin, norepinephrine, and dopamine synthesis, and thus polygenic effects due to the accumulation of mutations in multiple genes may play a role in this population (Trefz, Maillot, Motzfeldt, & Schwarz, 2011; Vockley, Rinaldo, Bennett, Matern, & Vladutiu, 2000).

The findings in this patient population are different than reports of empirical treatment 5-L-methyltetrahydrofolate (5LMTHF) (Fava & Mischoulon, 2009; Jain & Jackson, 2012; Papakostas et al., 2012, 2014). Adjunctive treatment with 5LMTHF may improve depressive symptoms because of its role as a cofactor in monoamine metabolism (Ramaekers, 2004). This is different from our findings in cerebral folate deficiency (Serrano et al., 2012). Also, 5LMTHF is most helpful in those with an MTHFR polymorphism that is known to have a deleterious effect (Fava & Mischoulon, 2009). No such variants were identified in the 8 patients with CFD for whom we have completed gene testing. Follow up studies regarding MTHFR status in this cohort are forthcoming.

We continue to work to understand the biologic mechanism of our metabolomic findings. Treatment with 5LMTHF is not sufficient in these individuals, because of a deficit earlier in the folate metabolic pathway that produces bioactive metabolites. Furthermore, the administration of 5LMTHF will correct the CSF test to identify these individuals. While empirical treatment with folinic acid may seem appealing, this again is problematic with our current understanding. After folinic acid administration, definitive testing cannot be completed, and the full effects of treatment with folinic acid in an individual with CFD occur over years. This time frame is due to neuronal turnover and growth in the setting of availability of active folate metabolites needed for appropriate white matter structure.

In 2011 (Pan et al., 2011), we reported a patient with severe treatment refractory depression and suicidal behavior whose CSF revealed deficient neopterin, biopterin, 5-HIAA and HVA (Werner-Felmayer et al., 2002). His suicidal ideation and behavior improved with sapropterin replacement. At that time, we considered this a first report of a rare, unique presentation of BH4 deficiency presenting with isolated, severe psychiatric symptoms. In this cohort, eleven patients were found to have tetrahydrobiopterin deficiency with similar CSF profile. This is a known neurometabolic disorder with an available treatment, tetrahydrobiopterin replacement in the form of sapropterin. Tetrahydrobiopterin is essential to the production of serotonin, dopamine, melatonin, norepinephrine, epinephrine, nitrous oxide, and pain modulators. As would be expected, replacement of the deficient small molecule results in improvement

Twelve participants with abnormal serum acylcarnitine profiles were identified. We do hypothesize that treatment with riboflavin 100 mg po qd might benefit some of these patients and referred these patients for further evaluation of potential mitochondrial disease by medical genetics. In addition, we are studying cell lines from some to assess for any signature of dysfunction of energy metabolism. We do not have data yet for these individuals, there are reports of acylcarnitine profile abnormalities in major depressive disorder (Ahmed et al., 2018). Acylcarnitine metabolomic profiles inform clinically defined major depressive phenotypes (Ahmed et al., 2020).

Twenty participants were found to have serum amino acid abnormalities. Future directions could include deeper exploration of specific amino acid abnormalities in larger cohorts. Several recent articles implicate serum amino acids in depression (Leblhuber et al., 2021; Peplinska-Miaskowska, Wichowicz, Smoleński, Jablonska, & Kaska, 2022; Rahman et al., 2022). Baranyi et al. (2016) suggests that decreased systemic levels of certain amino acids could compromise mTOR, leading to decreased energy metabolism in patients with depression. This is of interest, given that mTOR has been hypothesized to play a role in the mechanism of ketamine (Krystal, Abdallah, Sanacora, Charney, & Duman, 2019). While we did not have data on treatment intervention in these 32 patients, these findings indicate potential avenues of pursuit for patients with treatment refractory depression.

It is clear that our findings of neurometabolic abnormalities are complex, and our findings do not yet address causation. However, they point to numerous possible additional opportunities for study. Abnormalities in these pathways could indicate underlying unrecognized systemic problems. For example, the fundamental defect may be de novo purine and pyrimidine synthesis, leading to reduction of formyl-methionine-tRNA availability for mitochondrial protein synthesis. Under stress from any number of causes, cellular ATP is released through pannexin/P2 × 7 channels in the cell membrane, creating extracellular ATP (eATP) which is a potent damage associated molecular pattern and ligand for purinergic receptors. The loss of intracellular ATP to create eATP signaling depletes intracellular ATP pools, which in turn forces an increase in de novo purine synthesis, which requires more methylene-THF for dTMP synthesis, and more formyl-THF (or leucovorin) for IMP synthesis. Methyl-THF is used to regenerate methionine from homocysteine. An intramitochondrial deficit of methionine or 1-carbon intermediates could lead to impairment of mitochondrial biogenesis with resultant mitochondria fragmentation. In addition, if redox conditions stay unfavorable, then insufficient IMP is converted to GMP and GTP. Depletion of GTP pools causes depletion of biopterin because GTP cyclohydrolase does not have adequate GTP. Also, when the redox state is worsening, mitochondria do not make enough UMP for cellular pyrimidine needs. This happens because mitochondrial dihydroorotate dehydrogenase is unable to efficiently oxidize dihydroorotic acid to orotic acid needed for UMP synthesis.

We cannot determine if the metabolite abnormalities are primary or secondary. However, our patients had no other overt clinical disease and thus secondary deficiencies related to other systemic conditions are unlikely. Applications of next generation sequencing technologies (i.e. whole exome or genome sequencing) on a focused, homogeneous patient population with treatment refractory depression may provide further insight into genetic components of the metabolic findings in our patients. After further evaluation and characterization of a larger population of patients, animal models of biochemical abnormalities will be an important next step.

### Limitations

Though the majority of patients in this study were recruited by advertisement through a research registry, four participants were clinically referred. Therefore, the possibility of an ascertainment bias cannot be excluded. This was a relatively small sample of mostly Caucasian patients, and there was no treatment trial, but rather follow-up on clinical management of our participants. Continued treatment, as usual, resulted in medication changes during the course of the study. Specifically, in three cases of CFD there were concomitant medication changes in addition to folinic acid supplementation. Preliminary experience with treatment has been promising; however, long term outcomes remain to be determined.

Only 16 out of 20 participants with cerebral folate deficiency complied with treatment with folinic acid. Similarly, only seven out of 11 participants with low tetrahydrobiopterin intermediates and five out of 20 with borderline low tetrahydrobiopterin intermediates complied with treatment with sapropterin.

CSF testing is required to determine the presence of cerebral folate deficiency and subsequent need for treatment. Review of the CSF metabolic profile may direct the diagnosis and avoids missing other metabolic disorders that may be present. 5-L-methyltetrahydrofolate is the treatment specific to methylenetetrahydrofolate deficiency (Knowles, Morris, & Walter, 2016), but in the majority of cerebral folate deficiency cases, folinic acid is preferable because of its position earlier in the folate metabolism pathway. Dihydrofolate reductase converts folic acid to dihydrofolate and then to tetrahydrofolate (online Supplementary Fig. S3). Folinic acid (5-formyl-tetrahydrofolate) is converted to 5-,10- methenyltetrahydrofolate, then 5-,10- methylenetetrahydrofolate, and, eventually, to 5-methyltetrahydrofolate. Folinic acid, therefore, allows the availability of active metabolites in the folate pathway in individuals with cerebral folate deficiency that is not caused by methylenetetrahydrofolate deficiency. Furthermore, folinic acid has therapeutic effect in the setting of folate receptor autoantibodies, though there is controversy surrounding the assessment of these autoantibodies (Ramaekers et al., 2014).

Folinic acid should be given by prescription. Doses of 1–2 mg/kg may be administered with prescription strength folinic acid. Treatment with either folinic acid or 5MTHF will preclude testing after administration because they correct deficient CSF 5MTHF levels. We anticipate a clinical response should be expected in patients with treatment refractory depression and low CSF 5MTHF. Experience with more severe neurologic disorder associated with CFD (Molero-Luis et al., 2015) indicates that the response may take several months but the full effect of treatment may take 1–3 years. This may be related to neuronal growth and turnover in an environment where folate metabolites are newly available. Folic acid is not recommended because of the potentially low activity of dihydrofolate reductase in cerebral folate deficiency, and because of its tight binding to folate receptor alpha that results in reduced cerebral transport of 5-methyltetrahydrofolate. Folinic acid treatment in our patients did have some side effects, which included facial flushing and increased anxiety with initiation of treatment. It is important to note that vitamin B12 acts as a cofactor in this pathway. Blood folate and B12 levels should be evaluated in all patients with treatment refractory depression. The best option for patients with treatment refractory depression for whom metabolic disorder is suspected is consultation with a biochemical geneticist.

Future directions include repeat metabolic testing, including repeat testing after intervention. It would be beneficial to compare treatment refractory patients to treatment responsive patients, but this was beyond the scope of this study.

### Summary

Our findings indicate a need for further evaluation of the role of neurometabolic abnormalities in treatment refractory depression. The remarkably high incidence of actionable abnormalities, even in the absences of a demonstrable single gene disorder, and evidence in support of symptom improvement with treatment strongly support the need for larger studies. Historically, a diagnosis of a metabolic disorder has been considered in the context of a family history of a known disorder, or symptoms that are exacerbated by significant physiologic stresses (e.g. fever, fasting, surgeries), especially with multi-system involvement (Walterfang, Bonnot, & Mocellin, 2013), neither of which were present in our patient population. Our findings, when further replicated suggest that defined neurometabolic disorders may contribute to treatment-refractory psychiatric disorder even without other systemic illness. An important future question is whether early identification and treatment of an underlying metabolic abnormality early in the course of psychiatric illness would prevent long-term emotional and cognitive complications. These findings suggest that the identification of both genetic and secondary disorders of metabolism contributing to psychiatric illness may allow repurposing of currently approved orphan drugs for the treatment of severe treatment refractory depression in some cases.

## Supplementary material

For supplementary material accompanying this paper visit https://doi.org/10.1017/S0033291722003233. click here to view supplementary material

## References

[ref1] Ahmed, A. T., Frye, M. A., Rush, A. J., Biernacka, J. M., Craighead, W. E., & McDonald, W. M., … Mood Disorders Precision Medicine Consortium. (2018). Mapping depression rating scale phenotypes onto research domain criteria (RDoC) to inform biological research in mood disorders. Journal of Affective Disorders, 238, 1–7. 29807322 10.1016/j.jad.2018.05.005 PMC6374030

[ref2] Ahmed, A. T., MahmoudianDehkordi, S., Bhattacharyya, S., Arnold, M., Liu, D., & Neavin, D., … Mood Disorders Precision Medicine Consortium. (2020). Acylcarnitine metabolomic profiles inform clinically-defined major depressive phenotypes. Journal of Affective Disorders, 264, 90–97. 32056779 10.1016/j.jad.2019.11.122 PMC7024064

[ref4] Anglin, R. E., Garside, S. L., Tarnopolsky, M. A., Mazurek, M. F., & Rosebush, P. I. (2012). The psychiatric manifestations of mitochondrial disorders: A case and review of the literature. Journal of Clinical Psychiatry, 73(4), 506–512. 22579150 10.4088/JCP.11r07237

[ref5] Anonymous (2014). The burden of depression. Nature, 515(7526), 163. 10.1038/515163a 25391922

[ref6] Asberg, M., & Forslund, K. (2000). Neurobiological aspects of suicidal behaviour. International Review of Psychiatry, 12(1), 62–74.

[ref7] Asberg, M., Traskman, L., & Thoren, P. (1976). 5-HIAA in the cerebrospinal fluid: A biochemical suicide predictor? Archives General Psychiatry, 33(10), 1193–1197. 10.1001/archpsyc.1976.01770100055005 971028

[ref8] Axelrod, J. (1972). Biogenic amines and their impact in psychiatry. Seminars in Psychiatry, 4(3), 199–210. 4680221

[ref9] Baranyi, A., Amouzadeh-Ghadikolai, O., von Lewinski, D., Rothenhäusler, H. B., Theokas, S., Robier, C., … Meinitzer, A. (2016). Branched-chain amino acids as new biomarkers of major depression-a novel neurobiology of mood disorder. PLoS ONE, 11(8), e0160542. 27490818 10.1371/journal.pone.0160542 PMC4973973

[ref10] Beck, A. T., Schuyler, D., & Herman, I. (1974). Development of suicidal intent scales. In A. T. Beck, H. L.P. Resnick & D. J. Lettieri (Eds.), The prediction of suicide (pp. 45–56). Bowie, MD: Charles Press.

[ref11] Beck, A. T., Ward, C. H., Mendelson, M., Mock, J., & Erbaugh, J. (1961). An inventory for measuring depression. Archives of General Psychiatry, 4, 561–571. 13688369 10.1001/archpsyc.1961.01710120031004

[ref12] Bottiglieri, T., Hyland, K., Laundy, M., Godfrey, P., Carney, M. W., Toone, B. K., & Reynolds, E. H. (1992). Folate deficiency, biopterin and monoamine metabolism in depression. Psychological Medicine-London, 22, 871. 10.1017/s0033291700038447 1283223

[ref13] Centers for Disease Control and Prevention. (2022). Center for Disease Control and Prevention Data and Statistical Fatal Injury Report.

[ref3] Clarke, D. & Kuhl E. (2014). DSM-5 cross-cutting symptom measures: a step towards the future of psychiatric care? *World Psychiatry*, (3), 314–316. doi: 10.1002/wps.20154 PMC4219074 25273306

[ref14] Cohen, B. H., Chinnery, P. F., & Copeland, W. C. (2010). POLG-related disorders. In R. A. Pagon, M. P. Adam, H. H. Ardinger, S. E. Wallace, A. Amemiya, L. J. H. Bean, T. D. Bird, C. R. Dolan, C. T. Fong, R. J. H. Smith & K. Stephens (Eds.), GeneReviews (pp. 1997–2010). Seattle, WA: University of Washington. 20301791

[ref15] Fava, M. (2003). Diagnosis and definition of treatment-resistant depression. Biological Psychiatry, 53(8), 649–659. 12706951 10.1016/s0006-3223(03)00231-2

[ref16] Fava, M., & Mischoulon, D. (2009). Folate in depression: Efficacy, safety, differences in formulations, and clinical issues. The Journal of Clinical Psychiatry, 70(5), 12–17. 10.4088/JCP.8157su1c.03 19909688

[ref17] Garcia-Cazorla, A., Quadros, E. V., Nascimento, A., Garcia-Silva, M. T., Briones, P., Montoya, J., & Ramaekers, V. T. (2008). Mitochondrial diseases associated with cerebral folate deficiency. Neurology, 70(16), 1360–1362. 18413591 10.1212/01.wnl.0000309223.98616.e4

[ref18] Hyland, K., Shoffner, J., & Heales, S. J. (2010). Cerebral folate deficiency. Journal of Inherited Metabolic Disorders, 33(5), 563–570. 10.1007/s10545-010-9159-6 20668945

[ref19] Jain, R., & Jackson, W. C. (2012). Beyond the resistance: How novel neurobiological understandings of depression may lead to advanced treatment strategies. The Journal of Clinical Psychiatry, 73(11), e30. 23218168 10.4088/JCP.12020wc3

[ref20] Knowles, L., Morris, A. A., & Walter, J. H. (2016). Treatment with mefolinate (5-methyltetrahydrofolate), but not folic acid or folinic acid, leads to measurable 5–methyltetrahydrofolate in cerebrospinal fluid in methylenetetrahydrofolate reductase deficiency. Journal of Inherited Metabolic Disease, 29, 103–107. 10.1007/8904_2016_529 PMC5059208 26898294

[ref21] Krystal, J. H., Abdallah, C. G., Sanacora, G., Charney, D. S., & Duman, R. S. (2019). Ketamine: A paradigm shift for depression research and treatment. Neuron, 101(5), 774–778. 30844397 10.1016/j.neuron.2019.02.005 PMC6560624

[ref22] Leblhuber, F., Geisler, S., Ehrlich, D., Steiner, K., Reibnegger, G., Fuchs, D., & Kurz, K. (2021). Repetitive transcranial magnetic stimulation in the treatment of resistant depression: Changes of specific neurotransmitter precursor amino acids. Journal of Neural Transmission, 128(8), 1225–1231. 34244826 10.1007/s00702-021-02363-7 PMC8321996

[ref23] Molero-Luis, M., Serrano, M., O'Callaghan, M. M., Sierra, C., Pérez-Dueñas, B., García-Cazorla, A., & Artuch, R. (2015). Clinical, etiological and therapeutic aspects of cerebral folate deficiency. Expert Review of Neurotherapeutics, 15(7), 793–802. 26092490 10.1586/14737175.2015.1055322

[ref24] Morris, G., & Berk, M. (2015). The many roads to mitochondrial dysfunction in neuroimmune and neuropsychiatric disorders. BioMed Central Medicine, 13, 68. 10.1186/s12916-015-0310-y PMC4382850 25889215

[ref25] NIMH (1992). *NIMH Genetics Initiative. Family Interview for Genetic Studies (FIGS)*. Rockville, MD: National Institute of Mental Health.

[ref26] Pan, L., Martin, P., Zimmer, T., Segreti, A., Kassiff, S., McKain, B. W., &… Vockley, J. (2016). Neurometabolic disorders: Potentially treatable abnormalities in patients with treatment refractory depression and suicidal behavior. The American Journal of Psychiatry, 174(1), 42–50. 27523499 10.1176/appi.ajp.2016.15111500 PMC10171090

[ref27] Pan, L., McKain, B. W., Madan-Khetarpal, S., Mcguire, M., Diler, R., Perel, J., & Brent, D. A. (2011). GTP-cyclohydrolase deficiency responsive to sapropterin and 5-HTP supplementation: Relief of treatment-refractory depression and suicidal behaviour. BMJ Case Reports. doi:10.1136/bcr.03.2011.3927 PMC3116226 22691588

[ref28] Pan, L., & Vockley, J. (2013). Neuropsychiatric symptoms in inborn errors of metabolism: Incorporation of genomic and metabolomic analysis into therapeutics and prevention. Current Genetic Medicine Reports, 1(1), 65–70. 23525354 10.1007/s40142-012-0004-0 PMC3603703

[ref29] Papakostas, G. I., Shelton, R. C., Zajecka, J. M., Bottiglieri, T., Roffman, J., Cassiello, C., & … Fava, M. (2014). Effect of adjunctive L-methylfolate 15 mg among inadequate responders to SSRIs in depressed patients who were stratified by biomarker levels and genotype: Results from a randomized clinical trial. The Journal of Clinical Psychiatry, 1–9. 10.4088/JCP.13m08947 24813065

[ref30] Papakostas, G. I., Shelton, R. C., Zajecka, J. M., Etemad, B., Rickels, K., Clain, A., … Fava, M. (2012). L-methylfolate as adjunctive therapy for SSRI-resistant major depression: Results of two randomized, double-blind, parallel-sequential trials. The American Journal of Psychiatry, 169(12), 1267–1274. 23212058 10.1176/appi.ajp.2012.11071114

[ref31] Peplinska-Miaskowska, J., Wichowicz, H., Smoleński, R., Jablonska, P., & Kaska, L. (2022). The comparison of nucleotide metabolites and amino acids patterns in patients with eating disorders, with and without symptoms of depression. Nucleosides, Nucleotides & Nucleic Acids, 41(3), 333–341. 10.1080/15257770.2022.2028827 35076345

[ref32] Posner, K., Oquendo, M. A., Gould, M., Stanley, B., & Davies, M. (2007). Columbia classification algorithm of suicide assessment (C-CASA): Classification of suicidal events in the FDA's pediatric suicidal risk analysis of antidepressants. The American Journal of Psychiatry, 164, 1035–1043. 17606655 10.1176/appi.ajp.164.7.1035 PMC3804920

[ref33] Poznanski, E. O., & Mokros, H. B. (1996). Children's depression rating scale revised *(*CDRS-R*)*. Los Angeles: Western Psychological Services.

[ref34] Quijada-Fraile, P., O'Callaghan, M., Martín-Hernández, E., Montero, R., Garcia-Cazorla, A., Martinez de Aragon, A., … Artuch, R. (2014). Follow-up of folinic acid supplementation for patients with cerebral folate deficiency and Kearns-Sayre syndrome. Orphanet Journal of Rare Disorders, 9, 217. 10.1186/s13023-014-0217-2 PMC4302586 25539952

[ref35] Rahman, S., Shanta, A. A., Daria, S., Nahar, Z., Shahriar, M., Qusar, M. S., … Islam, M. R. (2022). Increased serum resistin but not G-CSF levels are associated in the pathophysiology of major depressive disorder: Findings from a case-control study. PLoS ONE, 17(2), e0264404. 35213631 10.1371/journal.pone.0264404 PMC8880862

[ref36] Ramaekers, V. T. (2004). Cerebral folate deficiency. Developmental Medicine and Child Neurology, 46, 843–851. 15581159 10.1017/s0012162204001471

[ref37] Ramaekers, V. T., Thöny, B., Sequeira, J. M., Ansseau, M., Philippe, P., Boemer, F., & Quadros, E. V. (2014). Folinic acid treatment for schizophrenia associated with folate receptor autoantibodies. Molecular Genetics and Metabolism, 113(4), 307–314. 25456743 10.1016/j.ymgme.2014.10.002

[ref38] Reynolds, W. M. (1987). Suicidal ideation questionnaire: Professional manual. Odessa, FL: Psychological Assessment Resources.

[ref39] Sedel, F. (2013). Cerebral folate deficiency. Presentation at behavioural and psychiatric aspects of inborn errors of metabolism. Paris, France: Orphan Europe Academy.

[ref40] Serrano, M., Perez-Duenas, B., Montoya, J., Ormazabal, A., & Artuch, R. (2012). Genetic causes of cerebral folate deficiency: Clinical, biochemical, and therapeutic aspects. Drug Discovery Today, 17(23–24), 1299–1306. 22835503 10.1016/j.drudis.2012.07.008

[ref41] Souery, D., Papkostas, G. I., & Trivedi, M. H. (2006). Treatment resistant depression. Journal of Clinical Psychiatry, 67(6), 16–22. 16848672

[ref42] Trefz, F., Maillot, F., Motzfeldt, K., & Schwarz, M. (2011). Adult phenylketonuria outcome and management. Molecular Genetics and Metabolism, 104, S26–S30. 21944883 10.1016/j.ymgme.2011.08.025

[ref43] Vockley, J., Rinaldo, P., Bennett, M. J., Matern, D., & Vladutiu, G. D. (2000). Synergisitic heterozygosity: Disease resulting from multiple partial defects in one or more metabolic pathways. Molecular Genetics and Metabolism, 71(1–2), 10–18. 11001791 10.1006/mgme.2000.3066

[ref44] Walterfang, W., Bonnot, O., & Mocellin, R. (2013). The neuropsychiatry of inborn errors of metabolism. Journal of Inherited Metabolic Disorders, 36, 687–702. 10.1007/s10545-013-9618-y 23700255

[ref45] Werner-Felmayer, G., Golderer, G., & Werner, E. R. (2002). Tetrahydrobiopterin biosynthesis, utilization and pharmacological effects. Current Drug Metabolism, 3(2), 159–173. 12003348 10.2174/1389200024605073

