# Supplementary material for: Metabolomic disorders: confirmed presence of potentially treatable abnormalities in patients with treatment refractory depression and suicidal behavior
Source: Psychol Med. 2022 Nov 4;53(13):6046–54. doi: 10.1017/S0033291722003233 (PMC10520591; doi:10.1017/S0033291722003233)
Supplement: Supplementary file 1 [file S0033291722003233sup.zip › S0033291722003233sup001.docx]

**Supplemental Figure 3:** Summary of folate pathway. Folinic acid (5-formyl-THF) enters the pathway without the action of DHFR and contributes active metabolites. 5-Methyl-THF is a distal metabolite.


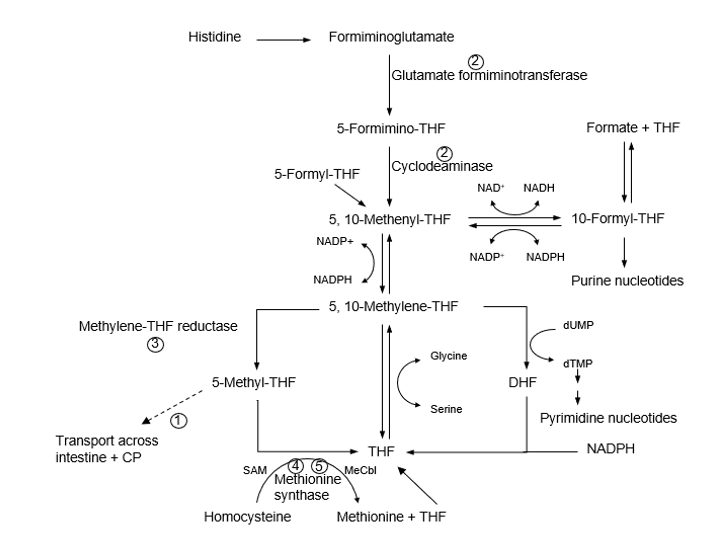


DHFR= dihydrofolate reductase, THF= tetrahydrofolate, DHF= dihydrofolate, dUMP= deoxy-uridine phosphate, dTMP= deoxy-thymidine phosphate, CP= choroid plexus, SAM= S-adenosylmethionine, NADP= nicotinamide adenine dinucleotide phosphate.
