# Supplementary material for: Metabolomic disorders: confirmed presence of potentially treatable abnormalities in patients with treatment refractory depression and suicidal behavior
Source: Psychol Med. 2022 Nov 4;53(13):6046–54. doi: 10.1017/S0033291722003233 (PMC10520591; doi:10.1017/S0033291722003233)
Supplement: Supplementary file 1 [file S0033291722003233sup.zip › S0033291722003233sup002.docx]

**Supplemental Figure 4:** Summary of the tetrahydrobiopterin pathway


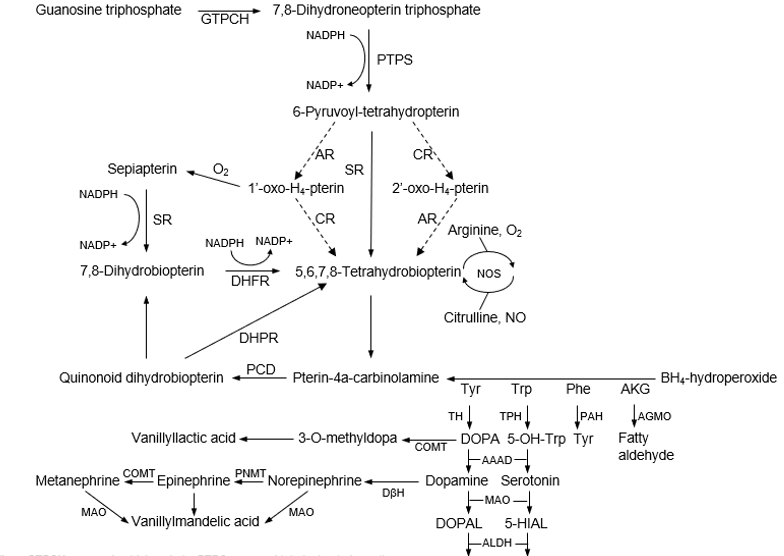


5-HIAA HVA

**GTPCH=** guanosine triphosphate, **PTPS=** pyruvoyl tetrahydropterin synthase, **SR=** sepiapterin reductase, **DHFR=** dihydrofolate reductase, **DHPR=** dihydropteridine reductase, **PCD=** pterin carbinolamine 4-dehydratase, **CR=** carbonyl reductase, **AR=** aldose reductase, **AAAH=** aromatic amino acid hydroxylase, **AGMO=** alkyl-glycerol monooxygenase, **NOS=** nitric oxide synthase, **AAAD=** aromatic amino acid decarboxylase, **ALDH=** aldehyde dehydrogenase, **DβH=** Dopamine-β-hydroxylase, **AKG=** alkylglycerol, **5-HIAA=** 5-hydroxyindoleacetic acid, **HVA=** homovanillic acid, **5-HIAL=** 5-hydroxyindoleacetaldehyde, **MAO=** monoamine oxidase, **DOPA=** levodopa, **5-OH-Trp=** 5-hydroxy tryptophan, **Tyr=** tyrosine, **Trp=** tryptophan, **Phe=** phenylalanine, **TH=** tyrosine hydroxylase, **TPH=** tryptophan hydroxylase, **PAH=** phenylalanine hydroxylase, **DOPAL=** dihydroxyphenylacetic acid, **COMT=** catechol-O-methyltransferase, **PNMT=** phenylethanolamine n-methyltransferase.
