# Supplementary material for: Metabolomic disorders: confirmed presence of potentially treatable abnormalities in patients with treatment refractory depression and suicidal behavior
Source: Psychol Med. 2022 Nov 4;53(13):6046–54. doi: 10.1017/S0033291722003233 (PMC10520591; doi:10.1017/S0033291722003233)
Supplement: Supplementary file 1 [file S0033291722003233sup.zip › S0033291722003233sup003.docx]

**Supplemental Table 3**: Characterization of Depression

| **Cerebral Folate Deficiency-Depressed** | ***Number of Depressed Episodes** | *** Age at Onset of Depression (years)** | ***Level of Impairment: 1=Impaired, 2=Incapacitated** | ***Duration of Longest Episode (weeks)** | ***Number of Suicide Attempts** | **Comorbid Disorder** | **History of Bipolar Disorder yes/no** | **Family History of MDD yes/no** | **Family History of BP yes/no** | **Number of Medication Trials** | **ECT yes/no:** |
| --- | --- | --- | --- | --- | --- | --- | --- | --- | --- | --- | --- |
| 1 | 90 | 14 | 2 | 12 | 0 | Substance Use Disorder | no | no | yes | 3 | no |
| 2 | 2 | 15 | 2 | 600 | 1 | PTSD and Anxiety | no | yes | yes | 6 | no |
| 3 | 1 | 9 | 2 | 468 | 1 | Anxiety | no | yes | no | >10 | yes |
| 4 | 2 | 12 | 2 | 468 | 5 | PTSD, Anxiety, and ADHD | no | yes | no | 8 | no |
| 5 | 1 | 26 | 2 | 208 | 4 | PTSD | no | no | yes | 4 | yes |
| 6 | >100 | 9 | 2 | 50 | 0 | PTSD, ADHD, and Substance Use Disorder | yes | yes | yes | 9 | no |
| 7 | 2 | 16 | 2 | 312 | 0 | Substance Use Disorder | no | yes | no | 4 | no |
| 8 | 2 | 12 | 2 | 72 | 0 | Anxiety | no | no | no | 4 | no |
| 9 | 2 | 13 | 2 | 156 | 5 | PTSD | no | yes | no | >10 | no |
| 10 | 1 | 13 | 2 | 1144 | 1 | Anxiety and Anorexia Nervosa | no | yes | yes | >10 | yes |
| 11 | 2 | 11 | 1 | 72 | 0 | Anxiety | no | yes | no | 4 | no |
| 12 | 1 | 11 | 2 | 312 | >10 | Anxiety, Anorexia Nervosa, and OCD | yes | yes | no | >10 | yes |
| 13 | 5 | 13 | 2 | 260 | 0 | Anxiety | no | yes | no | >10 | no |
| 14 | 1 | 11 | 2 | 362 | 0 | Anxiety, Eating Disorder NOS, and PTSD | no | yes | yes | 3 | yes |
| 15 | 1 | 8 | 2 | 2236 | 1 | PTSD, Asperger's, and past Substance Use Disorder | no | yes | no | >10 | yes |
| 16 | 1 | 7 | 2 | 1248 | 0 | Anxiety and Eating Disorder NOS | no | yes | no | >10 | yes |
| 17 | 1 | 30 | 2 | 884 | 0 | PTSD and Anxiety | no | yes | yes | >10 | yes |
| 18 | 2 | 17 | 2 | 364 | 0 | Anxiety, ADHD, Asperger's, and OCD | no | yes | no | >10 | no |
| 19 | 1 | 4 | 2 | 1300 | 3 | PTSD and Anxiety | no | yes | no | 9 | no |
| 20 | 1 | 20 | 2 | 28 | 1 | none | no | no | no | 3 | no |
|  |  |  |  |  |  |  |  |  |  |  |  |
| **Low Tetrahydrobiopterin Intermediates Depressed** | ***Number of Depressed Episodes** | *** Age at Onset of Depression (years)** | ***Level of Impairment: 1=Impaired, 2=Incapacitated** | ***Duration of Longest Episode (weeks)** | ***Number of Suicide Attempts** | **Comorbid Disorder** | **History of Bipolar Disorder yes/no** | **Family History of MDD yes/no** | **Family History of BP yes/no** | **Number of Medication Trials** | **ECT yes/no:** |
| 14 | 1 | 11 | 2 | 362 | 0 | Anxiety, Eating Disorder NOS, and PTSD | no | yes | yes | 3 | yes |
| 15 | 1 | 8 | 2 | 2236 | 1 | PTSD, Asperger's, and past Substance Use Disorder | no | yes | no | >10 | yes |
| 16 | 1 | 7 | 2 | 1248 | 0 | Anxiety and Eating Disorder NOS | no | yes | no | >10 | yes |
| 21 | 1 | 10 | 2 | 260 | 1 | Anxiety, Eating Disorder NOS, and OCD | no | no | no | >10 | yes |
| 22 | 1 | 15 | 2 | 832 | 3 | Anxiety, Eating Disorder NOS, and OCD | no | yes | no | 6 | yes |
| 23 | 1 | 11 | 2 | 520 | 0 | Anxiety, Eating Disorder NOS, and PTSD | no | yes | no | >10 | no |
| 24 | 4 | 16 | 2 | 260 | 1 | Anxiety and ADHD | no | no | no | >10 | no |
| 25 | 1 | 3 | 2 | 2080 | 0 | PTSD and Anxiety | no | yes | no | 5 | no |
| 26 | 1 | 3 | 2 | 988 | >10 | PTSD and Anxiety | no | yes | no | >10 | no |
| 27 | 2 | 14 | 2 | 364 | 0 | Phelan McDermid Syndrome with speech delay and intellectual disability, Anxiety | yes | yes | yes | >10 | yes |
| 28 | 1 | 11 | 2 | 780 | 1 | PTSD and Anxiety | yes | no | no | >10 | no |
|  |  |  |  |  |  |  |  |  |  |  |  |
| **Borderline Low Tetrahydrobiopterin Intermediates Depressed** | ***Number of Depressed Episodes** | *** Age at Onset of Depression (years)** | ***Level of Impairment: 1=Impaired, 2=Incapacitated** | ***Duration of Longest Episode (weeks)** | ***Number of Suicide Attempts** | **Comorbid Disorder** | **History of Bipolar Disorder yes/no** | **Family History of MDD yes/no** | **Family History of BP yes/no** | **Number of Medication Trials** | **ECT yes/no:** |
| 29 | 1 | 13 | 2 | 208 | 2 | Anxiety, Eating Disorder NOS, and OCD | no | yes | yes | 5 | yes |
| 30 | 1 | 16 | 2 | 988 | 0 | Anxiety | yes | no | no | >10 | yes |
| 31 | 1 | 12 | 2 | 208 | 3 | Anxiety | yes | yes | no | 8 | no |
| 32 | 1 | 17 | 2 | 1300 | 0 | Anxiety and ADHD | yes | yes | yes | >10 | no |
| 33 | 1 | 25 | 2 | 1716 | 0 | Anxiety | yes | yes | no | >10 | no |
| 34 | 1 | 15 | 2 | 1040 | 3 | Anxiety and OCD | yes | yes | no | >10 | no |
| 35 | 1 | 13 | 1 | 1404 | 0 | Anxiety | no | no | no | >10 | no |
| 36 | 1 | 15 | 1 | 520 | 0 | Anxiety and ADHD | no | yes | no | >10 | no |
| 37 | 3 | 14 | 2 | 780 | 0 | Anxiety | yes | yes | no | >10 | yes |
| 38 | 1 | 12 | 1 | 468 | 3 | Asperger's | no | no | no | 8 | no |
| 39 | 1 | 11 | 2 | 312 | 1 | Anxiety and PTSD | no | yes | no | 8 | no |
| 40 | 1 | 13 | 1 | 156 | 0 | Anxiety and Autism | no | no | no | 5 | no |
| 41 | 1 | 3 | 2 | 2756 | 0 | Anxiety, PTSD, and Substance Use Disorder | no | yes | no | >10 | yes |
| 42 | 5 | 5 | 2 | 1040 | 1 | Anxiety, and past Substance Use Disorder | no | yes | no | >10 | no |
| 43 | 5 | 34 | 2 | 60 | 0 | Anxiety and past Anorexia | no | yes | no | >10 | no |
| 44 | 1 | 12 | 2 | 208 | 1 | Anxiety | no | no | no | 5 | no |
| 45 | 5 | 16 | 2 | 24 | 0 | none | no | yes | no | >10 | no |
| 46 | 1 | 12 | 2 | 1567 | 0 | Anxiety | no | yes | no | 5 | no |
| 47 | 1 | 12 | 2 | 1352 | 10 | PTSD and Psychosis NOS, and past Substance Use Disorder | yes | yes | yes | >10 | no |
| 48 | 1 | 14 | 2 | 1248 | 2 | Anxiety and Borderline Personality Disorder | yes | yes | no | >10 | yes |
|  |  |  |  |  |  |  |  |  |  |  |  |
| **Abnormal Serum Acylcarnitine Profile-Depressed** | ***Number of Depressed Episodes** | *** Age at Onset of Depression (years)** | ***Level of Impairment: 1=Impaired, 2=Incapacitated** | ***Duration of Longest Episode (weeks)** | ***Number of Suicide Attempts** | **Comorbid Disorder** | **History of Bipolar Disorder yes/no** | **Family History of MDD yes/no** | **Family History of BP yes/no** | **Number of Medication Trials** | **ECT yes/no:** |
| 1 | 90 | 14 | 2 | 12 | 0 | Substance Use Disorder | no | no | yes | 3 | no |
| 46 | 1 | 12 | 2 | 1567 | 0 | Anxiety | no | yes | no | 5 | no |
| 47 | 1 | 12 | 2 | 1352 | 10 | PTSD and Psychosis NOS, past Substance Use Disorder | yes | yes | yes | >10 | no |
| 49 | 1 | 17 | 2 | 1092 | 0 | PTSD, past Substance Use Disorder | yes | yes | yes | >10 | no |
| 50 | 2 | 13 | 2 | 364 | 0 | Anxiety | no | yes | no | 6 | yes |
| 51 | 1 | 5 | 2 | 1092 | 0 | OCD and PTSD | no | yes | no | 4 | no |
| 52 | 1 | 13 | 2 | 364 | 1 | Anxiety and ADHD | no | yes | no | 9 | no |
| 53 | 1 | 13 | 1 | 156 | 1 | Anxiety , ADHD, and Substance Use Disorder | no | no | no | >10 | no |
| 54 | 1 | 15 | 2 | 104 | 0 | Anxiety and ADHD | no | no | no | 7 | no |
| 55 | 2 | 14 | 2 | 520 | 0 | None | no | yes | no | >10 | no |
| 56 | 2 | 8 | 2 | 1040 | 3 | Anxiety , ADHD, and Substance Use Disorder | yes | no | no | >10 | yes |
| 57 | 1 | 13 | 2 | 416 | 1 | Anxiety and OCD | no | yes | no | 5 | no |
|  |  |  |  |  |  |  |  |  |  |  |  |
|  |  |  |  |  |  |  |  |  |  |  |  |
| **Abnormal Serum Amino Acids-Depressed** | ***Number of Depressed Episodes** | *** Age at Onset of Depression (years)** | ***Level of Impairment: 1=Impaired, 2=Incapacitated** | ***Duration of Longest Episode (weeks)** | ***Number of Suicide Attempts** | **Comorbid Disorder** | **History of Bipolar Disorder yes/no** | **Family History of MDD yes/no** | **Family History of BP yes/no** | **Number of Medication Trials** | **ECT yes/no:** |
| 6 | >100 | 9 | 2 | 50 | 0 | PTSD, ADHD, and Substance Use Disorder | yes | yes | yes | 9 | no |
| 7 | 2 | 16 | 2 | 312 | 0 | Substance Use Disorder | no | yes | no | 4 | no |
| 10 | 1 | 13 | 2 | 1144 | 1 | Anxiety and Anorexia Nervosa | no | yes | yes | >10 | yes |
| 17 | 1 | 30 | 2 | 884 | 0 | PTSD and Anxiety | no | yes | yes | >10 | yes |
| 23 | 1 | 11 | 2 | 520 | 0 | Anxiety, Eating Disorder NOS, and PTSD | no | yes | no | >10 | no |
| 25 | 1 | 3 | 2 | 2080 | 0 | PTSD and Anxiety | no | yes | no | 5 | no |
| 28 | 1 | 11 | 2 | 780 | 1 | PTSD and Anxiety | yes | no | no | >10 | no |
| 30 | 1 | 16 | 2 | 988 | 0 | Anxiety | yes | no | no | >10 | yes |
| 31 | 1 | 12 | 2 | 208 | 3 | Anxiety | yes | yes | no | 8 | no |
| 43 | 5 | 34 | 2 | 60 | 0 | Anxiety and past Anorexia | no | yes | no | >10 | no |
| 45 | 5 | 16 | 2 | 24 | 0 | none | no | yes | no | >10 | no |
| 55 | 2 | 14 | 2 | 520 | 0 | None | no | yes | no | >10 | no |
| 58 | 1 | 10 | 2 | 520 | 0 | Anxiety | no | yes | no | 7 | no |
| 59 | 1 | 16 | 2 | 364 | 3 | Anxiety and PTSD | yes | yes | no | >10 | no |
| 60 | 2 | 11 | 2 | 208 | 3 | Anxiety and Eating Disorder NOS | no | no | no | 6 | no |
| 61 | 1 | 18 | 2 | 1456 | 0 | Anxiety and OCD | no | yes | no | >10 | no |
| 62 | 1 | 13 | 2 | 1404 | 5 | Anxiety, past Substance Use Disorder, and past Eating Disorder NOS | no | yes | no | >10 | yes |
| 63 | 1 | 16 | 2 | 1716 | 0 | Anxiety, OCD, and ADHD | no | yes | no | >10 | yes |
| 64 | 6 | 5 | 2 | 156 | 0 | Anxiety and ADHD | no | yes | no | 4 | no |
| 65 | 1 | 15 | 2 | 1196 | 0 | Anxiety | yes | yes | yes | 7 | no |
|  |  |  |  |  |  |  |  |  |  |  |  |
|  |  |  |  |  |  |  |  |  |  |  |  |
| **No Metabolic Disorder-Depressed** | ***Number of Depressed Episodes** | *** Age at Onset of Depression (years)** | ***Level of Impairment: 1=Impaired, 2=Incapacitated** | ***Duration of Longest Episode (weeks)** | ***Number of Suicide Attempts** | **Comorbid Disorder** | **History of Bipolar Disorder yes/no** | **Family History of MDD yes/no** | **Family History of BP yes/no** | **Number of Medication Trials** | **ECT yes/no:** |
| 66 | >100 | 21 | 2 | 104 | 1 | Anxiety and past Substance Use Disorder | yes | no | no | >10 | no |
| 67 | 2 | 5 | 2 | 468 | 0 | Anxiety | no | yes | no | 5 | no |
| 68 | 2 | 11 | 2 | 208 | 0 | Anxiety and OCD | no | no | no | 4 | no |
| 69 | 15 | 8 | 2 | 12 | 2 | Anxiety | no | yes | yes | 10 | no |
| 70 | 1 | 10 | 2 | 416 | 2 | Anxiety | no | yes | no | 6 | no |
| 71 | 1 | 14 | 2 | 104 | 4 | Anxiety, OCD and Autism Spectrum Disorder | no | yes | no | >10 | no |
| 72 | 5 | 8 | 2 | 156 | 0 | Anxiety | no | yes | no | >10 | no |
| 73 | 1 | 9 | 2 | 520 | 1 | Anxiety and Eating Disorder NOS | no | yes | no | >10 | no |
| 74 | 1 | 14 | 2 | 364 | 0 | Anxiety and ADHD | no | yes | no | >10 | no |
| 75 | 2 | 15 | 1 | 156 | 2 | Anxiety | no | no | no | 9 | no |
| 76 | 1 | 14 | 1 | 1248 | 1 | Anxiety and ADHD | no | yes | no | >10 | no |
| 77 | 5 | 14 | 2 | 156 | 1 | Anxiety, Eating Disorder NOS, and PTSD | yes | yes | no | 6 | no |
| 78 | 6 | 9 | 2 | 468 | 1 | PTSD and Anxiety | no | yes | no | 6 | no |
| 79 | 1 | 12 | 2 | 884 | 2 | none | no | yes | yes | 8 | no |
| 80 | 5 | 9 | 2 | 728 | 0 | none | no | yes | no | 7 | no |
| 81 | 1 | 23 | 2 | 572 | 0 | Anxiety | no | no | no | 10 | no |
| 82 | 2 | 6 | 2 | 364 | 0 | none | no | yes | no | 8 | no |
| 83 | 1 | 13 | 2 | 364 | 4 | PTSD and Psychosis | no | yes | no | >10 | yes |
| 84 | 1 | 14 | 1 | 1042 | 0 | PTSD, Anxiety, and Substance Use Disorder | no | yes | yes | 7 | no |
| 85 | 1 | 7 | 2 | 780 | 0 | none | no | yes | no | 4 | no |
| 86 | 4 | 13 | 2 | 36 | 1 | Anxiety | no | yes | no | 4 | no |
| 87 | 1 | 14 | 2 | 416 | 0 | Non-Verbal learning Disability | yes | no | yes | >10 | no |
| 88 | 1 | 20 | 1 | 572 | 2 | Anorexia Nervosa | no | no | no | >10 | yes |
| 89 | 1 | 20 | 2 | 468 | 0 | Anxiety and OCD | yes | no | no | >10 | yes |
| 90 | 1 | 13 | 2 | 936 | 3 | Anxiety and OCD | no | yes | no | >10 | yes |
| 91 | >20 | 16 | 2 | 1050 | 0 | ADHD | no | yes | yes | 5 | no |
| 92 | 1 | 12 | 1 | 572 | 0 | Anxiety | no | no | no | 5 | no |
| 93 | 1 | 16 | 1 | 104 | 4 | Anxiety | no | yes | no | 5 | no |
| 94 | 1 | 4 | 2 | 1196 | 2 | Anxiety and PTSD | no | no | no | 7 | no |
| 95 | 1 | 2 | 2 | 832 | 0 | Anxiety and Autism Spectrum Disorder | no | yes | no | 5 | no |
| 96 | 1 | 11 | 1 | 208 | 0 | Anxiety and ADHD | no | yes | no | 6 | no |
| 97 | 1 | 15 | 2 | 36 | 0 | none | no | yes | no | 3 | no |
| 98 | 1 | 13 | 2 | 260 | 0 | Anxiety | no | no | yes | >10 | no |
| 99 | 1 | 9 | 2 | 364 | 4 | Anxiety, ADHD, and Anorexia Nervosa | yes | yes | no | >10 | no |
| 100 | 1 | 7 | 1 | 312 | 0 | Anxiety and Tourette's | no | yes | no | 8 | no |
| 101 | 6 | 6 | 2 | 156 | 1 | Anxiety | no | yes | no | 4 | no |
| 102 | 1 | 7 | 2 | 364 | 2 | Anxiety and OCD | no | yes | no | 5 | no |
| 103 | 1 | 9 | 2 | 370 | 5 | Anxiety and ADHD | yes | yes | yes | >10 | no |
| 104 | 1 | 14 | 1 | 104 | 0 | none | no | no | no | >10 | no |
| 105 | 1 | 12 | 2 | 260 | 2 | Anxiety, OCD, and Autism Spectrum Disorder | no | no | no | 3 | no |
| 106 | 1 | 12 | 1 | 208 | 0 | Anxiety | no | no | yes | 4 | no |
| 107 | 1 | 12 | 2 | 208 | 0 | Anxiety and ADHD | no | yes | no | >10 | no |
| 108 | 1 | 11 | 2 | 156 | >10 | Anxiety and ADHD | yes | yes | no | 6 | no |
| 109 | 1 | 6 | 2 | 624 | 9 | Anxiety and ADHD | no | yes | no | >10 | no |
| 110 | 5 | 16 | 2 | 260 | 0 | Anxiety | no | yes | no | 9 | no |
| 111 | 1 | 10 | 2 | 468 | 0 | Anxiety and OCD | no | no | no | 5 | no |
| 112 | 1 | 12 | 2 | 884 | 0 | Anxiety | yes | no | no | >10 | yes |
| 113 | 1 | 14 | 2 | 780 | 0 | Anxiety, ADHD, and OCD | no | yes | no | >10 | no |
| 114 | 1 | 16 | 2 | 1040 | 0 | Anxiety | no | yes | no | >10 | no |
| 115 | >90 | 12 | 2 | 26 | 0 | Anxiety | yes | yes | no | >10 | no |
| 116 | 5 | 12 | 2 | 104 | 0 | Anxiety | yes | yes | no | >10 | no |
| 117 | 1 | 8 | 2 | 988 | 2 | Anxiety and PTSD | no | yes | no | 9 | yes |
| 118 | 1 | 5 | 2 | 3276 | 0 | Anxiety and Asperger's | no | yes | yes | >10 | no |
| 119 | 3 | 9 | 2 | 1560 | 1 | Anxiety and OCD | no | no | no | >10 | yes |
| 120 | 1 | 16 | 2 | 1092 | 0 | Anxiety | yes | yes | no | >10 | yes |
| 121 | 1 | 5 | 2 | 1092 | 1 | Anxiety, PTSD, Bulimia, Schizoaffective Disorder | no | no | no | >10 | yes |
| 122 | 1 | 13 | 2 | 260 | 0 | Anxiety | no | no | no | 4 | no |
| 123 | 1 | 5 | 2 | 780 | 3 | Substance Use Disorder | no | yes | no | 7 | no |
| 124 | 1 | 4 | 2 | 1092 | 5 | Anxiety, OCD, and Substance Use Disorder | no | yes | no | >10 | no |
| 125 | 5 | 19 | 2 | 208 | 0 | Anxiety and ADHD | no | yes | no | >10 | no |
| 126 | 5 | 16 | 2 | 780 | 1 | Anxiety | no | yes | no | >10 | yes |
| 127 | 1 | 5 | 2 | 936 | 0 | Anxiety and OCD | no | yes | yes | >10 | no |
| 128 | 1 | 5 | 2 | 2288 | 3 | Anxiety, PTSD, and OCD | no | yes | no | >10 | yes |
| 129 | 1 | 15 | 1 | 156 | 0 | Anxiety | no | no | no | 6 | no |
| 130 | 1 | 12 | 2 | 260 | 1 | Anxiety | no | yes | no | 9 | no |
| 131 | 1 | 14 | 2 | 260 | 2 | Anxiety | no | no | yes | >10 | no |
| 132 | 1 | 16 | 2 | 520 | 0 | Anxiety and Substance Use Disorder | no | yes | no | >10 | yes |
| 133 | 3 | 17 | 1 | 26 | 0 | Anxiety | yes | no | no | 4 | no |
| 134 | 1 | 12 | 2 | 364 | 0 | Anxiety | no | yes | no | >10 | yes |
| 135 | 1 | 22 | 2 | 52 | 0 | Anxiety, PTSD, and Substance Use Disorder | no | yes | no | >10 | no |
| 136 | 1 | 6 | 2 | 1040 | 2 | Anxiety, PTSD, ADHD, and OCD | no | no | yes | >10 | no |
| 137 | 1 | 8 | 2 | 520 | 1 | Anxiety, PTSD, past Eating Disorder NOS | no | yes | no | >10 | no |
| 138 | 1 | 23 | 2 | 936 | 1 | Anxiety and Schizoaffective Disorder | no | no | no | >10 | yes |
| 139 | 5 | 9 | 2 | 104 | 0 | PTSD and Borderline PD | no | yes | no | 6 | no |
| 140 | 1 | 5 | 2 | 1560 | 0 | Anxiety and OCD | no | yes | no | >10 | yes |
| 141 | 5 | 16 | 2 | 312 | 0 | Anxiety, PTSD, and ADHD | no | yes | no | >10 | no |

PTSD=Post Traumatic Stress Disorder, ADHD=Attention Deficit Hyperactivity Disorder, OCD= Obsessive Compulsive Disorder

*Assessed from Family Interview for Genetic Studies (FIGS) reports.
