# Supplementary material for: Metabolomic disorders: confirmed presence of potentially treatable abnormalities in patients with treatment refractory depression and suicidal behavior
Source: Psychol Med. 2022 Nov 4;53(13):6046–54. doi: 10.1017/S0033291722003233 (PMC10520591; doi:10.1017/S0033291722003233)
Supplement: Supplementary file 1 [file S0033291722003233sup.zip › S0033291722003233sup004.docx]

**Supplemental Table 4**: Depressed Participants Findings and Treatment Results

| **Cerebral Folate Deficiency-Depressed** | **Metabolic Findings:** | **CSF 5-MTHF Levels nmol/L (40-120)** | **Serum folate levels ng/mL (>5)** | **CSF Tetrahydrobiopterin Levels nmol/L (10-30)** | **Psychiatric Medications at Initial Visit** | **Initial SIQ** | **Initial BDI** | **Psychiatric Medications at Post-Treatment Visit** | **Post SIQ** | **Post BDI** | | **Time lapse between initial and post treatment self-reports (weeks)** |
| --- | --- | --- | --- | --- | --- | --- | --- | --- | --- | --- | --- | --- |
| 1 | Low CSF 5MTHF and abnormal acylcarnitine profile | 36 | >24.0 | 17 | none | 8 | 25 | Folinic acid | 4 | 21 | | 10 |
| 2 | Low CSF 5MTHF and low HVA | 40 | 6.1 | 14 | none | 13 | 22 | Folinic acid | 8 | 15 | | 7 |
| 3 | Low CSF 5MTHF and low HVA | 39 | >24.0 | 32 | Citalopram, Risperidone, Amitriptyline and Desyrel | 36 | 25 | Folinic acid, Amitriptyline, Desyrel, 5 HTP, and Carbidopa | 17 | 14 | | 36 |
| 4 | Low CSF 5MTHF, low 5HIAA, and low HVA | 37 | >24.0 | 17 | Desyrel | 61 | 29 | Folinic Acid, and Desyrel | 45 | 29 | | 8 |
| 5 | Low CSF 5MTHF | 39 | 12.2 | 25 | Zolpidem and Vilazodone | 70 | 37 | Folinic Acid, Zolpidem, and Vilazodone | 58 | 36 | | 11 |
| 6 | Low CSF 5MTHF, low 5HIAA, and low HVA; and serum amino acid elevated taurine and proline | 36 | >24.0 | 14 | Lioresal, Gabapentin, and Tramedol | 23 | 33 | Folinic Acid, Lioresal, Gabapentin, and Tramedol | 18 | 17 | | 20 |
| 7 | Low CSF 5MTHF and low 5HIAA; serum amino acid elevated alanine and taurine | 15 | >24.0 | 15 | Fluoxetine | 14 | 24 | Folinic Acid and Fluoxetine | 13 | 13 | | 15 |
| 8 | Low CSF 5MTHF and low HVA | 32 | 18.3 | 14 | Sertraline, Carbidopa, and Lamotrigine | 41 | 28 | Folinic Acid, Sertraline, Carbidopa, and Lamotrigine | 20 | 16 | | 7 |
| 9 | Low CSF 5MTHF | 35 | 14.4 | 23 | Sertraline | 67 | 43 | Folinic Acid | 15 | 0 | | 80 |
| 10 | Low CSF 5MTHF, and serum amino acid elevation in alanine | 24 | >24.0 | 16 | Trazodone and Duloxetine | 22 | 30 | Folinic acid, Trazodone and Duloxetine | 17 | 19 | | 5 |
| 11 | Low CSF 5MTHF | 37 | >24.0 | 14 | Venlafaxine and Bupropion | 35 | 14 | folinic acid, Venlafaxine and Bupropion | 11 | 15 | | 5 |
| 12 | Low CSF 5MTHF and 5HIAA | 33 | 22.6 | 14 | Clonazepam, Venlafaxine, Quetiapine, and Oxcarbazepine | 61 | 33 | Folinic acid, Clonazepam, Venlafaxine, Quetiapine, and Oxcarbazepine | 48 | 27 | | 5 |
| 13 | Low CSF 5MTHF and 5HIAA | 31 | >24.0 | 14 | Vilazodone, and Hydroxyzine | 47 | 35 | Folinic Acid, Vilazodone, and Hydroxyzine | 14 | 9 | | 8 |
| 14 | Low CSF 5MTHF, low tetrahydrobiopterin, low 5HIAA, and low HVA | 31 | >24.0 | 8 | Sertraline, Escitalopram, Lisdexamfetamine, and Hydroxyzine | 47 | 40 | Folinic Acid, 5HTP, Carbidopa, Sapropterin, Lisdexamfetamine, and Hydroxyzine | 14 | 12 | | 52 |
| 15 | Low CSF 5MTHF, and low tetrahydrobiopterin | 40 | 19.5 | 5 | none | 40 | 22 | Sapropterin and Levomefolic acid | 14 | 4 | | 28 |
| 16 | Low CSF 5MTHF, and low tetrahydrobiopterin | 32 | >24.0 | 9 | Bupropion, Methylphenidate, and Alprazolam | 60 | 55 | Sapropterin, Folinic Acid, Bupropion, Methylphenidate, and Alprazolam | 50 | 49 | | 28 |
| 17 | Low CSF 5MTHF, and serum amino acid elevation in alanine | 28 | >24.0 | 13 | Bupropion and Nefazodone | 59 | 39 | non-compliant to folinic acid | non-compliant | non-compliant | | non-compliant |
| 18 | Low CSF 5MTHF, 5HIAA, and HVA | 38 | >24.0 | 13 | none | 18 | 36 | non-compliant to folinic acid | non-compliant | non-compliant | | non-compliant |
| 19 | Low CSF 5MTHF, low 5HIAA, and low HVA | 40 | 17.6 | 13 | Lamotrigine, Escitalopram, Clonazepam, and Desyrel | 53 | 31 | non-compliant to folinic acid | non-compliant | non-compliant | | non-compliant |
| 20 | Low CSF 5MTHF | 37 | >24.0 | 19 | Bupropion | 20 | 20 | lost to follow up | lost | lost | | lost |
|  |  |  |  |  |  |  |  |  |  |  | |  |
| **Low Tetrahydrobiopterin Intermediates Depressed** | **Metabolic Findings:** | **CSF 5-MTHF Levels nmol/L (40-120)** | **Serum folate levels ng/mL (>5)** | **CSF Tetrahydrobiopterin Levels nmol/L (10-30)** | **Psychiatric Medications at Initial Visit** | **Initial SIQ** | **Initial BDI** | **Psychiatric Medications at Post-Treatment Visit** | **Post SIQ** | **Post BDI** | | **Time lapse between initial and post treatment self-reports (weeks)** |
| 14 | Low CSF 5MTHF, low tetrahydrobiopterin, low 5HIAA, and low HVA | 31 | >24.0 | 8 | Sertraline, Escitalopram, Lisdexamfetamine, and Hydroxyzine | 47 | 40 | Folinic Acid, 5HTP, Carbidopa, Sapropterin, Lisdexamfetamine, and Hydroxyzine | 14 | 12 | | 52 |
| 15 | Low CSF 5MTHF, and low tetrahydrobiopterin | 40 | 19.5 | 5 | none | 40 | 22 | Sapropterin and Levomefolic acid | 14 | 4 | | 28 |
| 16 | Low CSF 5MTHF, and low tetrahydrobiopterin | 32 | >24.0 | 9 | Bupropion, Methylphenidate, and Alprazolam | 60 | 55 | Sapropterin, Folinic Acid, Bupropion, Methylphenidate, and Alprazolam | 50 | 49 | | 28 |
| 21 | Low CSF tetrahydrobiopterin, 5HIAA, and HVA; | 112 | >24.0 | 9 | Venlafaxine, Sertraline, and Clonazepam | 83 | 46 | Sapropterin, Levomefolic acid, 5-Hyrdroxytryptophan, Carbidopa, Levothyroxine, Olanzapine, Clonazepam, and Hydroxyzine | 72 | 45 | | 52 |
| 22 | Low CSF tetrahydrobiopterin, 5HIAA, and HVA; | 103 | >24.0 | 9 | Fluoxetine, Aripiprazole, and Venlafaxine | 74 | 46 | Sapropterin, Venlafaxine, Aripiprazole, Lisdexamfetamine, and Clonazepam | 27 | 8 | | 60 |
| 23 | Low CSF tetrahydrobiopterin and HVA; and serum amino acid elevation in glutamine | 55 | 12.5 | 8 | Lamotrigine, Alprazolam, and D-Amphetamine | 58 | 53 | Sapropterin, Folinic Acid, Levomefolic acid, Carbidopa, Levothyroxine, Dextroamphetamine, Alprazolam and Lamotrigine | 37 | 41 | | 52 |
| 24 | Low CSF tetrahydrobiopterin | 73 | 14.2 | 6 | Alprazolam | 59 | 38 | Sapropterin and Alprazolam | 54 | 35 | | 6 |
| 25 | Low CSF tetrahydrobiopterin and serum amino acid elevation in glycine | 62 | >24.0 | 9 | Levothyroxine and Lorazepam | 15 | 34 | non-compliant to Sapropterin | non-compliant | non-compliant | | non-compliant |
| 26 | Low CSF tetrahydrobiopterin, 5HIAA, and HVA | 73 | >24.0 | 10 | Carbidopa | 72 | 44 | non-compliant to Sapropterin | non-compliant | non-compliant | | non-compliant |
| 27 | Low CSF tetrahydrobiopterin | 71 | >24.0 | 8 | Quetiapine, Lamotrigine, Olanzapine, and Lorazepam | 4 | 26 | Sapropterin, Quetiapine, Lamotrigine, Olanzapine, and Lorazepam | lost | lost | | lost to follow-up |
| 28 | Low CSF tetrahydrobiopterin and and serum amino acid elevation in alanine | 47 | >24.0 | 9 | Bupropion, Venlafaxine, Methylphenidate, Clonazepam, Zolpidem, and Mirtazapine | 18 | 20 | Sapropterin, Bupropion, Venlafaxine, Methylphenidate, Clonazepam, Zolpidem, and Mirtazapine | lost | lost | | lost to follow-up |
|  |  |  |  |  |  |  |  |  |  |  | |  |
| **Borderline Low Tetrahydrobiopterin Intermediates Depressed** | **Metabolic Findings:** | **CSF 5-MTHF Levels nmol/L (40-120)** | **Serum folate levels ng/mL (>5)** | **CSF Tetrahydrobiopterin Levels nmol/L (10-30)** | **Psychiatric Medications at Initial Visit** | **Initial SIQ** | **Initial BDI** | **Psychiatric Medications at Post-Treatment Visit** | **Post SIQ** | **Post BDI** | | **Time lapse between initial and post treatment self-reports (weeks)** |
| 29 | Lower CSF tetrahydrobiopterin, low 5HIAA and HVA | 61 | 20.9 | 12 | Duloxetine and Aripiprazole | 70 | 31 | Sapropterin, Levomefolic acid, Duloxetine and Aripiprazole | 52 | 30 | | 13 |
| 30 | Lower CSF tetrahydrobiopterin, low 5HIAA and HVA, and serum amino acid elevation in alanine | 97 | 12.2 | 11 | Clonazepam and Brexpiprazole | 34 | 49 | Sapropterin, Clonazepam and Brexpiprazole | 18 | 31 | | 52 |
| 31 | Lower CSF tetrahydrobiopterin, low 5HIAA and HVA, and serum amino acid elevation in citrulline | 62 | 19.2 | 11 | Aripiprazole, Lithium, and Lamotrigine | 56 | 14 | Sapropterin, Aripiprazole, Lithium, and Lamotrigine | 14 | 1 | | 27 |
| 32 | Lower CSF tetrahydrobiopterin and low 5HIAA | 55 | >24.0 | 11 | Venlafaxine, Trazodone, Clonazepam, Lisdexamfetamine, and Lamotrigine | 39 | 35 | Sapropterin, 5-Hydroxytryptophan, Carbidopa, Lamotrigine, Clonazepam, Lithium, Lisdexamfetamine, and Lurasidone | 16 | 6 | | 12 |
| 33 | Lower CSF tetrahydrobiopterin and low HVA | 89 | >24.0 | 11 | Lamotrigine, Quetiapine, Lorazepam, and Tranylcypromine | 4 | 21 | Sapropterin, 5-Hydroxytryptophan, Carbidopa, Lamotrigine, Clonazepam, Quetiapine, and Methylphenidate | 0 | 10 | | 8 |
| 34 | Lower CSF tetrahydrobiopterin, and low 5HIAA | 55 | 4.8 | 11 | Aripiprazole, Alprazolam, Lamotrigine, and Levothyroxine Sodium | 38 | 38 | non-compliant to Sapropterin | non-compliant | non-compliant | | non-compliant |
| 35 | Lower CSF tetrahydrobiopterin | 80 | 19.4 | 11 | Vilazodone, Methylphenidate, Zolpidem, and Lorazepam | 16 | 26 | non-compliant to Sapropterin | non-compliant | non-compliant | | non-compliant |
| 36 | Lower CSF tetrahydrobiopterin | 73 | 10.2 | 11 | Vortioxetine and D-Amphetamine | 50 | 30 | non-compliant to Sapropterin | non-compliant | non-compliant | | non-compliant |
| 37 | Lower CSF 5MTHF, and lower tetrahydrobiopterin | 42 | >24.0 | 11 | Clonazepam, Diazepam, and Gabapentin | non-compliant | 25 | non-compliant to folinic acid and Kuvan | non-compliant | non-compliant | | non-compliant |
| 38 | Lower CSF tetrahydrobiopterin | 64 | 13.3 | 11 | Venlafaxine, Lithium, Risperidone, and Trazodone | 53 | 33 | non-compliant to Sapropterin | non-compliant | non-compliant | | non-compliant |
| 39 | Lower CSF tetrahydrobiopterin | 71 | 14.6 | 12 | Duloxetine | 45 | 45 | non-compliant to Sapropterin | non-compliant | non-compliant | | non-compliant |
| 40 | Lower CSF tetrahydrobiopterin | 105 | >24.0 | 12 | Venlafaxine | 1 | 14 | non-compliant to Sapropterin | non-compliant | non-compliant | | non-compliant |
| 41 | Lower CSF tetrahydrobiopterin, and low 5HIAA | 53 | 19.3 | 12 | none | 61 | 52 | non-compliant to Sapropterin | non-compliant | non-compliant | | non-compliant |
| 42 | Lower CSF tetrahydrobiopterin, and low HVA | 57 | 24 | 11 | Bupropion and Diazepam | 59 | 43 | non-compliant to Sapropterin | non-compliant | non-compliant | | non-compliant |
| 43 | Lower tetrahydrobiopterin, and serum amino acid elevation in glycine | 49 | >24.0 | 11 | Vortioxetine, Lamotrigine, and Brexpiprazole | 44 | 38 | non-compliant to Sapropterin | non-compliant | non-compliant | | non-compliant |
| 44 | Lower CSF tetrahydrobiopterin and low 5HIAA | 78 | >24.0 | 12 | Fluoxetine, Lamotrigine, and Mirtazapine | 48 | 24 | non-compliant to Sapropterin | non-compliant | non-compliant | | non-compliant |
| 45 | Lower CSF tetrahydrobiopterin and low 5HIAA; serum amino acid elevations in alanine, taurine, proline, and glycine | 48 | 22 | 11 | Lisdexamfetamine, Desyrel, and Levothyroxine Sodium Sodium | 49 | 30 | non-compliant to Sapropterin | non-compliant | non-compliant | | non-compliant |
| 46 | Abnormal acylcarnitine profile, lower CSF tetrahydrobiopterin, and low 5HIAA | 56 | 18 | 11 | Bupropion and Sertraline | 24 | 12 | non-compliant to Sapropterin | non-compliant | non-compliant | | non-compliant |
| 47 | Abnormal acylcarnitine profile and lower CSF tetrahydrobiopterin | 60 | >24.0 | 12 | Lamotrigine, Clonazepam, and Fluoxetine | 33 | 35 | non-compliant to Sapropterin | non-compliant | non-compliant | | non-compliant |
| 48 | Lower CSF tetrahydrobiopterin, low CSF 5HIAA and HVA | 82 | >24.0 | 12 | Clonazepam, Gabapentin, and Vortioxetine | 27 | 31 | non-compliant to Sapropterin | non-compliant | non-compliant | | non-compliant |
|  |  |  |  |  |  |  |  |  |  |  | |  |
| **Abnormal Serum Acylcarnitine Profile-Depressed** | **Metabolic Findings:** | **CSF 5-MTHF Levels nmol/L (40-120)** | **Serum folate levels ng/mL (>5)** | **CSF Tetrahydrobiopterin Levels nmol/L (10-30)** | **Psychiatric Medications at Initial Visit** | **Initial SIQ** | **Initial BDI** |  |  |  | |  |
| 1 | Low CSF 5MTHF and abnormal acylcarnitine profile | 36 | >24.0 | 17 | none | 8 | 25 |  |  |  | |  |
| 46 | Abnormal acylcarnitine profile, lower CSF tetrahydrobiopterin, and low 5HIAA | 56 | 18 | 11 | Bupropion and Sertraline | 24 | 12 |  |  |  | |  |
| 47 | Abnormal acylcarnitine profile and lower CSF tetrahydrobiopterin | 60 | >24.0 | 12 | Lamotrigine, Clonazepam, and Fluoxetine | 33 | 35 |  |  |  | |  |
| 49 | Abnormal acylcarnitine profile | 92 | 10.2 | 24 | Gabapentin, Lithium, Lamotrigine, Clonazepam, and Olanzapine | 32 | 32 |  |  |  | |  |
| 50 | Abnormal acylcarnitine profile and low CSF 5HIAA | 109 | 19.3 | 18 | Levothyroxine Sodium Sodium, Ativan, Sertraline, Lurasidone, and Hydroxyzine | 36 | 29 |  |  |  | |  |
| 51 | Abnormal acylcarnitine profile, and low CSF 5HIAA | 68 | 17.6 | 29 | Citalopram, and Melatonin | 53 | 37 |  |  |  | |  |
| 52 | Abnormal acylcarnitine profile and Low creatine levels in urine/guan rat | 59 | >24.0 | 27 | Nortriptyline and Sertraline | 72 | 32 |  |  |  | |  |
| 53 | Abnormal acylcarnitine profile, and low CSF 5HIAA | 84 | 19.9 | 18 | Sertraline, Atomoxetine, and Venlafaxine | 74 | 44 |  |  |  | |  |
| 54 | Abnormal acylcarnitine profile, and low CSF 5HIAA and HVA | 48 | 13.4 | 15 | Bupropion, D-Amphetamine, and Fluoxetine | 56 | 31 |  |  |  | |  |
| 55 | Abnormal acylcarnitine profile, serum amino acid elevation in alanine, and low CSF 5HIAA and HVA | 74 | >24.0 | 21 | Vortioxetine, Clonazepam, and Aripiprazole | 33 | 23 |  |  | |  |  |
| 56 | Abnormal acylcarnitine profile, and low CSF 5HIAA | 65 | >24.0 | 35 | Sertraline, Divalproex sodium, and Gabapentin | 80 | 41 |  |  | |  |  |
| 57 | Abnormal acylcarnitine profile | 64 | >24.0 | 28 | Bupropion, Sertraline, and Clonazepam | 35 | 34 |  |  | |  |  |
|  |  |  |  |  |  |  |  |  |  |  | |  |
|  |  |  |  |  |  |  |  |  |  |  | |  |
| **Abnormal Serum Amino Acids-Depressed** | **Metabolic Findings:** | **CSF 5-MTHF Levels nmol/L (40-120)** | **Serum folate levels ng/mL (>5)** | **CSF Tetrahydrobiopterin Levels nmol/L (10-30)** | **Psychiatric Medications at Initial Visit** | **Initial SIQ** | **Initial BDI** |  |  |  | |  |
| 6 | Low CSF 5MTHF, low 5HIAA, and low HVA; and serum amino acid elevated taurine and proline | 36 | >24.0 | 14 | Lioresal, Gabapentin, and Tramedol | 23 | 33 |  |  |  | |  |
| 7 | Low CSF 5MTHF and low 5HIAA; serum amino acid elevated alanine and taurine | 15 | >24.0 | 15 | Fluoxetine | 14 | 24 |  |  |  | |  |
| 10 | Low CSF 5MTHF, and serum amino acid elevation in alanine | 24 | >24.0 | 16 | Trazodone and Duloxetine | 22 | 30 |  |  |  | |  |
| 17 | Low CSF 5MTHF, and serum amino acid elevation in alanine | 28 | >24.0 | 13 | Bupropion and Nefazodone | 59 | 39 |  |  |  | |  |
| 23 | Low CSF tetrahydrobiopterin and HVA; and serum amino acid elevation in glutamine | 55 | 12.5 | 8 | Lamotrigine, Alprazolam, and D-Amphetamine | 58 | 53 |  |  |  | |  |
| 25 | Low CSF tetrahydrobiopterin and serum amino acid elevation in glycine | 62 | >24.0 | 9 | Levothyroxine and Lorazepam | 15 | 34 |  |  |  | |  |
| 28 | Low CSF tetrahydrobiopterin and 5HIAA; and serum amino acid elevation in alanine | 47 | >24.0 | 9 | Bupropion, Venlafaxine, Methylphenidate, Clonazepam, Zolpidem, and Mirtazapine | 18 | 20 |  |  |  | |  |
| 30 | Lower CSF tetrahydrobiopterin, low 5HIAA and HVA, and serum amino acid elevation in alanine | 97 | 12.2 | 11 | Clonazepam and Brexpiprazole | 34 | 49 |  |  |  | |  |
| 31 | Lower CSF tetrahydrobiopterin, low 5HIAA and HVA, and serum amino acid elevation in citrulline | 62 | 19.2 | 11 | Aripiprazole, Lithium, and Lamotrigine | 56 | 14 |  |  |  | |  |
| 43 | Lower tetrahydrobiopterin, and serum amino acid elevation in glycine | 49 | >24.0 | 11 | Vortioxetine, Lamotrigine, and Brexpiprazole | 44 | 38 |  |  |  | |  |
| 45 | Lower CSF tetrahydrobiopterin and low 5HIAA; serum amino acid elevations in alanine, taurine, proline, and glycine | 48 | 22 | 11 | Lisdexamfetamine, Desyrel, and Levothyroxine Sodium Sodium | 49 | 30 |  |  |  | |  |
| 55 | Serum amino acid elevation in alanine, and low CSF 5HIAA and HVA | 74 | >24.0 | 21 | Vortioxetine, Clonazepam, and Aripiprazole | 33 | 23 |  |  |  | |  |
| 58 | Serum amino acid elevation in phenylalanine | 47 | 17.1 | 16 | Vortioxetine | 40 | 25 |  |  |  | |  |
| 59 | Serum amino acid elevation in alanine | 81 | 13.6 | 17 | Gabapentin and Vortioxetine | 71 | 33 |  |  |  | |  |
| 60 | Serum amino acid elevation in alanine, and low CSF 5HIAA and HVA | 94 | 13.4 | 15 | Venlafaxine and Lithium | 33 | 26 |  |  |  | |  |
| 61 | Serum amino acid elevation in alanine, and low CSF HVA | 70 | 15 | 20 | Diazepam, Escitalopram, and Clonazepam | 20 | 28 |  |  |  | |  |
| 62 | Serum amino acid elevation in alanine | 78 | 17.6 | 26 | Tranylcypromine and Levothyroxine Sodium | 51 | 39 |  |  |  | |  |
| 63 | Serum amino acid elevation in alanine, and low CSF 5HIAA and HVA | 91 | >24.0 | 16 | Clonazepam, Fluoxetine, and D-Amphetamine | 18 | 25 |  |  |  | |  |
| 64 | Serum amino acid elevation in alanine | 56 | 8.6 | 17 | None | 44 | 30 |  |  |  | |  |
| 65 | Serum amino acid elevation in alanine | 77 | 13.4 | 15 | none | 18 | 25 |  |  |  | |  |
|  |  |  |  |  |  |  |  |  |  |  | |  |
|  |  |  |  |  |  |  |  |  |  |  | |  |
| **No Metabolic Disorder-Depressed** | **Other Findings:** | **CSF 5-MTHF Levels nmol/L (40-120)** | **Serum folate levels ng/mL (>5)** | **CSF Tetrahydrobiopterin Levels nmol/L (10-30)** | **Psychiatric Medications at Initial Visit** | **Initial SIQ** | **Initial BDI** |  |  |  | |  |
| 66 | Fabry disease | 77 | 14.5 | 21 | Lithium, and Desyrel | 15 | 19 |  |  |  | |  |
| 67 | Low creatine levels in urine/guan rat | 55 | 13.4 | 17 | Aripiprazole, Diazepam, Concerta, Lamotrigine, and Bupropion | 15 | 38 |  |  |  | |  |
| 68 | Low CSF 5HIAA and HVA | 67 | >24.0 | 17 | Venlafaxine and Fluvoxamine | 22 | 24 |  |  |  | |  |
| 69 | Low CSF 5HIAA and HVA | 45 | 16.6 | 15 | Desvenlafaxine, Divalproex Sodium, and Aripiprazole | 17 | 26 |  |  |  | |  |
| 70 |  | 52 | 17.6 | 22 | none | 41 | 22 |  |  |  | |  |
| 71 | Low CSF 5HIAA | 49 | 15.4 | 15 | Bupropion, Risperidone, Escitalopram, and Lisdexamfetamine | 74 | 33 |  |  |  | |  |
| 72 | Low CSF HVA | 102 | 7.8 | 25 | Venlafaxine | 30 | 36 |  |  |  | |  |
| 73 | Low CSF 5HIAA and HVA | 98 | 14.5 | 22 | none | 59 | 36 |  |  |  | |  |
| 74 |  | 82 | 13 | 38 | Sertraline, Zolpidem, Lorazepam, and D-Amphetamine | 39 | 47 |  |  |  | |  |
| 75 | Low CSF 5HIAA and HVA | 53 | 11.5 | 24 | Citalopram and Aripiprazole | 20 | 22 |  |  |  | |  |
| 76 |  | 66 | 18.8 | 17 | none | 15 | 15 |  |  |  | |  |
| 77 |  | oxidized sample | 7.6 | oxidized sample | Fluoxetine | 58 | 30 |  |  |  | |  |
| 78 |  | 60 | 17.5 | 29 | Imipramine, Venlafaxinex, and Quetiapine Fumarate | 37 | 41 |  |  |  | |  |
| 79 | Low CSF 5HIAA and HVA | 46 | >24.0 | 25 | Sertraline and Quetiapine Fumarate | 59 | 33 |  |  |  | |  |
| 80 |  | 54 | >24.0 | 24 | Amitryptiline, Citalopram, Lovasal, and Levothyroxine Sodium | 12 | 12 |  |  |  | |  |
| 81 |  | 58 | 9.8 | 23 | Bupropion, Vilazodone, Temazepam, and Clonazepam | 11 | 19 |  |  |  | |  |
| 82 | Low CSF 5HIAA | 49 | 10.3 | 16 | Lamotrigine and Melatonin | 22 | 25 |  |  |  | |  |
| 83 | Low CSF 5HIAA | 59 | >24.0 | 15 | Clozapine | 88 | 49 |  |  |  | |  |
| 84 | Low CSF 5HIAA and HVA | 53 | >24.0 | 16 | Venlafaxine | 11 | 17 |  |  |  | |  |
| 85 |  | 59 | 18.6 | 27 | Clonazepam | 55 | 39 |  |  |  | |  |
| 86 |  | 51 | >24.0 | 17 | none | 20 | 22 |  |  |  | |  |
| 87 | Low CSF 5HIAA and HVA | 72 | 16.1 | 15 | Duloxetine and Lorazepam | 6 | 23 |  |  |  | |  |
| 88 | Low CSF 5HIAA | 65 | 23.2 | 19 | Desvenlafaxine, Olanzapine, and Clonazepam | 71 | 39 |  |  |  | |  |
| 89 | Low CSF HVA | 82 | >24.0 | 18 | none | 35 | 44 |  |  |  | |  |
| 90 |  | 102 | >24.0 | 13 | D-Amphetamine, Trazodone, Bupropion, Alprazolam, and Fluvoxamine | 54 | 34 |  |  |  | |  |
| 91 |  | 121 | 11.2 | 15 | Bupropion | 40 | 32 |  |  |  | |  |
| 92 |  | 99 | 18.9 | 14 | Sertraline and Lurasidone | 48 | 24 |  |  |  | |  |
| 93 | Low CSF 5HIAA | 72 | >24.0 | 33 | Venlafaxine and Clonazepam | 71 | 34 |  |  |  | |  |
| 94 | Low CSF 5HIAA | 92 | >24.0 | 14 | Vortioxetine and D-Amphetamine | 50 | 32 |  |  |  | |  |
| 95 |  | 74 | >24.0 | 29 | Venlafaxine | 20 | 25 |  |  |  | |  |
| 96 |  | 79 | 21 | 14 | Bupropion, Escitalopram, Aripriprazole, and Methylphenidate | 59 | 25 |  |  |  | |  |
| 97 | Low CSF 5HIAA and HVA | 67 | 19 | 15 | Bupropion and Fluoxetine | 44 | 19 |  |  |  | |  |
| 98 |  | 85 | >24.0 | 19 | Bupropion and Risperidone | 14 | 26 |  |  |  | |  |
| 99 | Low CSF 5HIAA and HVA | 77 | >24.0 | 24 | Bupropion and Naltrexone | 54 | 30 |  |  |  | |  |
| 100 | Low CSF 5HIAA and HVA | 60 | >24.0 | 13 | Fluoxetine | 69 | 54 |  |  |  | |  |
| 101 | Low CSF 5HIAA | 92 | >24.0 | 13 | Fluoxetine, Escitalopram, Venlafaxine, and Duloxetine | 62 | 25 |  |  |  | |  |
| 102 | Low CSF 5HIAA | 85 | 20.8 | 22 | Escitalopram and Aripiprazole | 56 | 17 |  |  |  | |  |
| 103 | Low CSF HVA | 78 | 17.3 | 14 | Methylphenidate and Risperidone | 73 | 40 |  |  |  | |  |
| 104 |  | 95 | >24.0 | 26 | Lithium, Doxycycline, Lamotrigine, and D-Amphetamine | 63 | 42 |  |  |  | |  |
| 105 | Low CSF 5HIAA | 98 | >24.0 | 22 | Fluoxetine | 32 | 25 |  |  |  | |  |
| 106 | Low CSF 5HIAA and HVA | 72 | >24.0 | 19 | Bupropione and Escitalopram | 66 | 40 |  |  |  | |  |
| 107 | Low CSF 5HIAA | 86 | 18.8 | 19 | Desvenlafaxine, Oxcarbazepine, and Buspirone | 6 | 11 |  |  |  | |  |
| 108 | Low CSF 5HIAA | 87 | 9.8 | 23 | Venlafaxine, Lurasidone, and Lithium | 35 | 18 |  |  |  | |  |
| 109 | Low CSF 5HIAA | 51 | >24.0 | 14 | Bupropione, Venlafaxine, Lamotrigine, Quetiapine, and Methylphenidate | 57 | 48 |  |  |  | |  |
| 110 | Low CSF 5HIAA | 87 | 12.3 | 18 | Diazepam and Venlafaxine | 27 | 26 |  |  |  | |  |
| 111 |  | 80 | 10.5 | 39 | None | 5 | 22 |  |  |  | |  |
| 112 | Low CSF 5HIAA and HVA | 72 | >24.0 | 19 | Lurasidone, D-Amphetamine, Clonazepam, Lamotrigine, and Trazodone | 42 | 40 |  |  |  | |  |
| 113 | Low CSF 5HIAA and HVA | 81 | >24.0 | 16 | Clonazepam and Duloxetine | 9 | 23 |  |  |  | |  |
| 114 | Low CSF 5HIAA and HVA | 69 | >24.0 | 19 | Bupropion, Atomoxetine, and Amitriptyline | 31 | 17 |  |  |  | |  |
| 115 | Low CSF 5HIAA and HVA | 72 | 22.8 | 14 | none | 9 | 5 |  |  |  | |  |
| 116 |  | 61 | 23.2 | 15 | Bupropion, Lamotrigine, Quetiapine, and Vilazodone | 6 | 23 |  |  |  | |  |
| 117 | Low CSF 5HIAA and HVA | 52 | 20.7 | 13 | Venlafaxine and Quetiapine | 47 | 29 |  |  |  | |  |
| 118 | Low CSF 5HIAA and HVA | 68 | >24.0 | 17 | Nortriptyline, Clonazepam, and D-Amphetamine | 16 | 21 |  |  |  | |  |
| 119 |  | 53 | >24.0 | 15 | Amitryptiline, Duloxetine, Fluoxetine, and Clonazepam | 22 | 41 |  |  |  | |  |
| 120 |  | 45 | >24.0 | 20 | Trazodone, Lamotrigine, and Lurasidone | 44 | 39 |  |  |  | |  |
| 121 |  | 85 | >24.0 | 23 | Haldol, and Alprazolam | 34 | 49 |  |  |  | |  |
| 122 | Low CSF 5HIAA | 50 | >24.0 | 22 | Aripiprazole, Bupropion, and Fluoxetine | 50 | 20 |  |  |  | |  |
| 123 |  | 71 | 8.5 | 27 | None | 31 | 27 |  |  |  | |  |
| 124 | Low CSF 5HIAA | 53 | 11.4 | 26 | Brexpiprazole and D-Amphetamine | 26 | 26 |  |  |  | |  |
| 125 |  | 67 | 18.4 | 26 | Duloxetine, Clonazepam, Zolidem, and Lisdexamfetamine | 40 | 45 |  |  |  | |  |
| 126 | Low CSF 5HIAA and HVA | 75 | >24.0 | 18 | Quetiapine, Clonazepam, and Levothyroxine Sodium | 21 | 28 |  |  |  | |  |
| 127 |  | 91 | 8.4 | 29 | None | 38 | 33 |  |  |  | |  |
| 128 |  | 102 | 15.1 | 20 | Trazodone, Clonazepam, and Duloxetine | 90 | 50 |  |  |  | |  |
| 129 |  | 73 | 17.5 | 23 | Duloxetine | 29 | 28 |  |  |  | |  |
| 130 | Low CSF 5HIAA | 68 | 10.8 | 19 | Haldol and Sertraline | 38 | 38 |  |  |  | |  |
| 131 | Low CSF 5HIAA and HVA | 88 | 19 | 29 | Fluoxetine | 51 | 24 |  |  |  | |  |
| 132 | Low CSF HVA | 62 | 12.3 | 13 | Trazodone and Olanzapine | 63 | 42 |  |  |  | |  |
| 133 | Low CSF 5HIAA and HVA | 72 | >24.0 | 20 | Venlafaxine | 38 | 36 |  |  |  | |  |
| 134 | Low CSF 5HIAA and HVA | 60 | >24.0 | 15 | Bupropion, Lamotrigine, and D-Amphetamine | 48 | 40 |  |  |  | |  |
| 135 | Low CSF 5HIAA | 91 | >24.0 | 16 | Clonazepam, Vortioxetine, and Methylphenidate | 36 | 38 |  |  |  | |  |
| 136 |  | 72 | 22.7 | 38 | Quetiapine, Escitalopram, and Lamotrigine | 56 | 34 |  |  |  | |  |
| 137 |  | 62 | >24.0 | 24 | Aripiprazole, Atomoxetine, and Vilazodone | 62 | 28 |  |  |  | |  |
| 138 | Low CSF HVA | 84 | >24.0 | 15 | Lamotrigine and Nortriptyline | 60 | 20 |  |  |  | |  |
| 139 | Low CSF 5HIAA and HVA | 56 | >24.0 | 17 | Duloxetine, Quetiapine, and Trazodone | 27 | 38 |  |  |  | |  |
| 140 | Low CSF 5HIAA and HVA | 58 | 15.1 | 19 | Sertraline | 39 | 36 |  |  |  | |  |
| 141 |  | 68 | 17.4 | 19 | Bupropion, Lisdexamfetamine, and Brexpiprazole | 44 | 39 |  |  |  | |  |

CSF = Cerebrospinal Fluid, 5-MTHF = 5-Methyltetrahydrofolate, SIQ = Suicide Ideation Questionnaire, BDI = Beck Depression Inventory
